# Impact of the Position of the Chemically Modified 5-Furyl-2′-Deoxyuridine Nucleoside on the Thrombin DNA Aptamer–Protein Complex: Structural Insights into Aptamer Response from MD Simulations

**DOI:** 10.3390/molecules24162908

**Published:** 2019-08-10

**Authors:** Preethi Seelam Prabhakar, Richard A. Manderville, Stacey D. Wetmore

**Affiliations:** 1 Department of Chemistry and Biochemistry, University of Lethbridge, 4401 University Drive West, Lethbridge, AL T1K 3M4, Canada; 2 Department of Chemistry and Toxicology, University of Guelph, Guelph, ON N1G 2W1, Canada

**Keywords:** Thrombin–binding aptamer, G-quadruplexes, chemical modification, fluorescent probes, computational modeling, protein binding.

## Abstract

Aptamers are functional nucleic acids that bind to a range of targets (small molecules, proteins or cells) with a high affinity and specificity. Chemically-modified aptamers are of interest because the incorporation of novel nucleobase components can enhance aptamer binding to target proteins, while fluorescent base analogues permit the design of functional aptasensors that signal target binding. However, since optimally modified nucleoside designs have yet to be identified, information about how to fine tune aptamer stability and target binding affinity is required. The present work uses molecular dynamics (MD) simulations to investigate modifications to the prototypical thrombin-binding aptamer (TBA), which is a 15-mer DNA sequence that folds into a G-quadruplex structure connected by two TT loops and one TGT loop. Specifically, we modeled a previously synthesized thymine (T) analog, namely 5-furyl-2′-deoxyuridine (5FurU), into each of the six aptamer locations occupied by a thymine base in the TT or TGT loops of unbound and thrombin bound TBA. This modification and aptamer combination were chosen as a proof-of-principle because previous experimental studies have shown that TBA displays emissive sensitivity to target binding based on the local environment polarity at different 5FurU modification sites. Our simulations reveal that the chemically-modified base imparts noticeable structural changes to the aptamer without affecting the global conformation. Depending on the modification site, 5FurU performance is altered due to changes in the local environment, including the modification site structural dynamics, degree of solvent exposure, stacking with neighboring bases, and interactions with thrombin. Most importantly, these changes directly correlate with the experimentally-observed differences in the stability, binding affinity and emissive response of the modified aptamers. Therefore, the computational protocols implemented in the present work can be used in subsequent studies in a predictive way to aid the fine tuning of aptamer target recognition for use as biosensors (aptasensors) and/or therapeutics.

## 1. Introduction

Aptamers are single-stranded DNA or RNA oligonucleotides that have the ability to recognize and bind targets with a high specificity and affinity [1,2]. Noncovalent intermolecular interactions allow aptamers to adopt a range of tertiary structures with diverse functions. Specifically, aptamers have been shown to fold into unique 3D-structures, possessing a combination of loops, stems, hairpins, pseudoknots, bulges and/or G-quadruplexes. As a result, aptamers have been designed to bind to a broad spectrum of target molecules including metals (Ag^+^ and Cu^2+^), dyes (malachite green), ligands (ATP, AMP, and arginamide), antibiotics (tetracycline, tobramycin, and neomycin), vitamins (B_12_), proteins (thrombin and cytochrome p450), hormones (progesterone), pesticides (organophosphorus), viruses (influenza H1N1) and cells (dendrites and liver carcinoma) [3]. This makes aptamers versatile tools for the detection of important biological targets for applications in imaging, sensing, diagnostics, and therapeutics [1,4,5]. 

**Figure 1:**
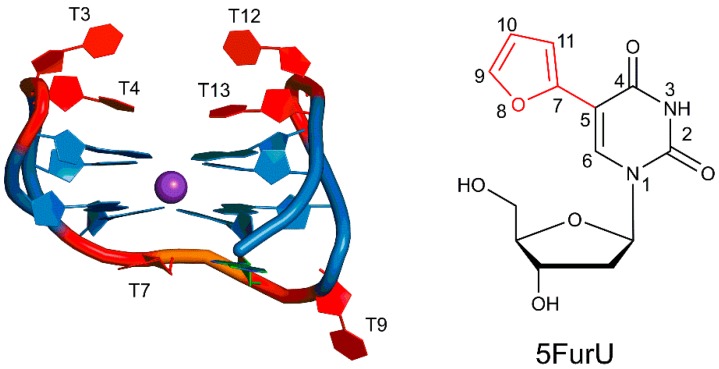
The thrombin-binding aptamer (TBA) antiparallel G-quadruplex, with T bases at six positions (3, 4, 7, 9, 12 and 13) highlighted in red and G-tetrads highlighted in blue (left), and the chemical structure of 5-furyl-2′-deoxyuridine (5FurU, right).

Among aptamers, those containing G-quadruplexes (GQ) have been found to be particularly promising for many key applications. G-quadruplexes are naturally-occurring nucleic acid motifs that are linked by nucleotide loops and stabilized by a monovalent cation(s). In general, GQ-forming oligonucleotides have attracted wide attention for their potential biological functions including roles in DNA replication, the regulation of transcription, telomere maintenance, and genome stability, as well as their potential applications in cancer therapies [6,7]. In terms of aptamers, GQ-forming oligonucleotides have shown promise in biomolecule detection through GQ-based fluorescent or luminescent probes [8,9]. The 15-mer thrombin-binding aptamer (TBA, 5′–GGTTGGTGTGGTTGG–3′) is perhaps the most studied G-quadruplex-forming aptamer. TBA possess anticoagulant properties by binding to the thrombin protein with high affinity and thereby inhibiting thrombin-catalyzed fibrin clot formation [10]. TBA folds into an antiparallel G-quadruplex, consisting of two G-quartets connected by three loops, namely a central TGT loop and two TT loops (Figure 1 and Figure S1A). The central cavity of the G-tetrad is occupied by one (K^+^) or more (Na^+^) metal ion(s). X-ray crystallographic studies of the TBA–thrombin complex have revealed that thrombin interacts with the two TT loops of TBA through a variety of noncovalent interactions [11,12]. Computational studies have also contributed to our understanding of TBA structure and thrombin affinity by investigating aptamer folding [13,14,15], structural dynamics [16,17], ion binding [18,19], and thrombin response [20]. Thus, TBA has proven to be an important model for understanding aptamer–target recognition.

Aptamers have the remarkable ability to tolerate a large number of chemical modifications to native nucleic acid backbones and/or nucleobases without losing significant activity. In fact, chemical modifications can enhance aptamer–target binding affinity or even broaden the target spectrum. For example, the 5-(indolyl-3-acetyl-3-amino-1-propenyl)-2′-deoxyuridine nucleobase analogue has demonstrated that chemical modification of the T4 position significantly improves the binding affinity of TBA to thrombin [21]. Additionally, the incorporation of a three-carbon spacer modification at T7 enhances the TBA–thrombin binding affinity [22]. However, not all chemical modifications improve aptamer stability and binding efficiency. For example, the incorporation of an unlocked nucleic acid modification at different TBA sites (G1, T9 and T12) negatively affects the GQ folding and anticoagulant properties [23].

In addition to chemical modifications that improve target binding, nucleic acid aptamers that contain a fluorophore have emerged as promising tools for fluorescent-based biosensors, which have potential applications in biomedical research and disease treatment [24,25]. In this approach, target–aptamer binding is signaled by changes in wavelength and fluorescence intensity arising from an alteration in the electronic environment surrounding the fluorophore upon target binding [26] and/or a change in the aptamer tertiary structure [27]. In fact, the measured change in fluorescence emission can directly reflect the extent of target binding, thereby allowing for the quantitative determination of the target in bioanalytical assays. In this light, several studies have focused on the use of internal fluorescent nucleobase mimics for monitoring TBA target binding [28,29,30,31]. For example, 8-heteroaryl-dG TBA probes at the G5 position exhibit fluorescence emission upon G-quadruplex formation [27,31]. Alternatively, azobenzene derivatives incorporated into the TGT loop behave as light-triggered molecular switches to assess aptamer stability and signal target binding [32]. Modifications to the TT loops have also been investigated with varying success [33,34,35]. For example, a modified aptamer containing α-thymidine at the T7 and T9 positions, and 5-nitroindole at G8 does not bind to thrombin [33]. Alternatively, the incorporation of a single 5-furyl-2′-deoxyuridine probe (5FurU, Figure 1) within any of the three unique TBA loops has demonstrated that the effectiveness of the probe is highly influenced by its position, with an increase in aptamer stability observed upon probe incorporation at positions 4, 9, or 13, but an enhancement in the target binding and fluorescence emission intensity upon probe incorporation at positions 3 or 12 [28].

Despite the promising utility of internal fluorescent nucleobase analogues to signal target binding, an optimal probe design has yet to be achieved, likely in part due to our current poor understanding of the factors that influence probe response. To better understand the behavior of internal fluorophores and aid the strategic optimization of new probes, molecular level information is required about the influence of the modification on the stability and structure of the unbound aptamer and target–aptamer complex. In this light, biochemical assays and biophysical (NMR, X-ray crystallography) studies have played critical roles in unveiling the molecular details of modified TBA–thrombin interactions [21,29,34,36,37,38,39,40,41,42,43,44,45,46,47,48,49]. For example, high-resolution X-ray crystal structures have been obtained for the modified TBA containing 5-(methyl-3-acetyl-3-amino-1-propenyl)-2′-deoxyuridine at the TT or TGT loops, both of which explain the seven-fold increase in the binding affinity relative to the native TBA based on stabilizing contacts of the T4-modified base at the aptamer–protein interface [21]. However, obtaining crystal structures for a wide variety of modifications is time and cost prohibitive. Alternatively, several computational studies on modified TBAs have provided useful insights into structural folding, stability and thrombin-binding affinities [27,30,44,50,51,52,53,54]. For example, computational studies on a modified TBA containing 5-nitroindole have provided evidence for a direct interaction between the modified central loop and thrombin [52], while investigations of a modified TBA containing 8-aryl-dG probes have explained that changes in the electronic structure of G5 in the G-tetrad that directly interacts with proteins correlates with changes in the stability of the aptamer–protein complex [27]. 

To better understand the factors affecting the performance of a modified aptamer containing an internal fluorescent probe, the present study used computational chemistry to investigate the structure and stability of the modified TBA and TBA–thrombin complex containing 5FurU as a prototypical example. Specifically, molecular dynamics (MD) simulations were used to systematically investigate the effects of the 5FurU chemical modification at each T loop position in TBA (3, 4, 7, 9, 12, and 13; Table 1). This modification was chosen because of the abundance of high-quality experimental data for all T modification positions including thermal melting curves, circular dichroism spectra, photophysical parameters, and thrombin binding affinities [28], which provide a unique platform to understand the performance of the modified base. By comparing the structural dynamics of the unbound aptamer and aptamer–protein complex for each modified TBA to the native (unmodified) aptamer, we reveal the impact of the modified T position and aptamer binding state on probe conformational flexibility, and we also determine how the modification changes intramolecular interactions within the aptamer and intermolecular interactions with the thrombin target. Through a comparison with previously published experimental biophysical data [28], our structural data rationalize the reported effects of the local environment of the nucleobase analogue on aptamer stability, target binding, and probe emissive properties. As a result, we have provided insight into the molecular features that can fine tune modified aptamer performance, including affording an enhanced fluorescence signal without negatively impacting aptamer stability or binding affinity, which will aid the design of new and improved nucleoside analogues for a range of biosensing applications.

## 2. Results and Discussion

### 2.1. 5FurU Modified TBA Maintains the G-Quadruplex Structure of Native TBA

To provide a basis for understanding the impact of thymine modifications on the structure and dynamics of an isolated (unbound) TBA, 0.5 μs MD simulations were initially performed on the native aptamer starting from the structure of the unbound aptamer extracted from a crystal structure corresponding to the human α-thrombin–aptamer complex (Protein Data Bank (PDB) ID: 4DII) [11]. The native TBA maintained a global G-quadruplex conformation throughout the MD simulation similar to the G-quadruplex in the crystal structure of the thrombin–TBA complex (Figures S1 and S2; Table S1) [11]. Specifically, the two G-tetrads were conserved (Table S2), with persistent Hoogsteen–edge interactions between neighboring guanines being mediated by two strong N1-H···O6 and N2-H···N7 hydrogen bonds that had optimal average donor–acceptor distances and angles (Tables S3 and S4). A K^+^ ion was positioned between the stacked G-tetrads that maintained perfect coordination with the eight O6 atoms of the G bases, with the average coordination distance falling between 2.7 and 2.8 Å (Table S5). 

The structural deviations in the loop bases in the native aptamer were greater across the simulation compared to those for the other bases (Figure S3), suggesting that loop residues were highly flexible. Nevertheless, the relative positions of the TGT and TT loop nucleobases match the thrombin–TBA crystal structure [11]. Specifically, T7 stacked with the G-tetrad G6 base (average interaction ~7 kcal/mol), G8 stacked with G10 (~7 kcal/mol), and T9 was exposed to the solvent (Figure S1). Among the TT loop bases, T3 and T12 were exposed to the solvent and exhibited a high degree of flexibility (Figures S1 and S3; Table 2), while the T4 and T13 were less flexible because of stacking interactions with neighboring bases (Table S6). Indeed, T4 stacked with G2, and T13 interacted with G11, with the average stacking interactions being ~−4.0 kcal/mol (Table S6). These observations agree with NMR studies that suggest the bases at positions 4 and 13 are involved in strong stacking interactions with the G-tetrad in the native TBA [55]. All of these stacking interactions between the nucleobases help stabilize the tertiary folding of the aptamer.

**Figure 2:**
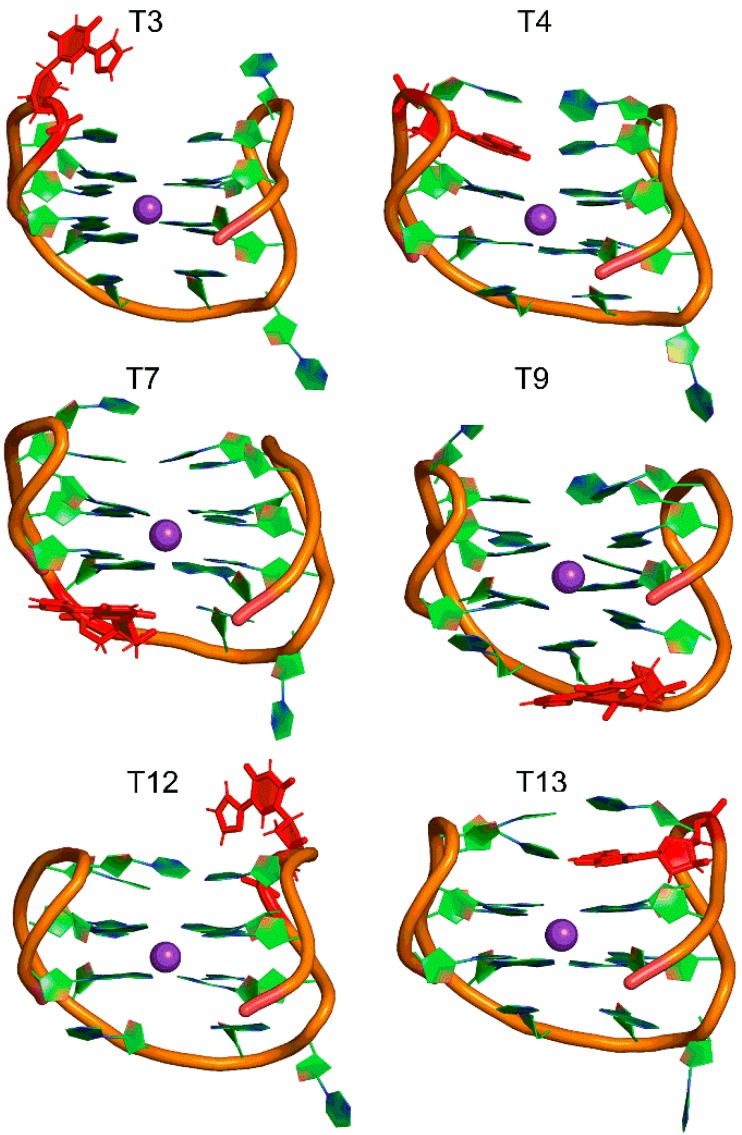
Representative MD structure of the unbound TBA containing 5FurU at positions T3, T4, T7, T9, T12 or T13 (red).

The structural dynamics of six modified TBAs containing a single 5FurU at one of the T locations (Table 1) were compared to the native TBA to understand the conformational preference of the unbound modified aptamer. Specifically, each modified TBA was built from the unmodified TBA structure by replacing the C5 methyl group of the T base at positions 3, 4, 7, 9, 12, or 13 with the 5-furyl moiety. For each modified TBA, no significant deviations in the overall backbone dynamics occurred throughout the MD simulations, and the average root-mean-square deviation (RMSD) with respect to the corresponding starting structure was close to the native aptamer regardless of the 5FurU position (Table S1 and Figure S2). Furthermore, the representative MD structures of each modified aptamer clearly depicted that the global conformation of the native TBA was maintained (Figure 2 and Figure S1), including the G-tetrads (Tables S2–S4), K^+^ coordination (Table S5) and stacked versus solvent-exposed positions of the nucleobases in the T-rich loops (Table 2 and Table S6). Furthermore, the modification did not significantly impact the trend in the dynamics of the individual nucleotides regardless of 5FurU incorporation site (Figure S3). This suggests that the incorporation of the 5FurU chemical modification did not disrupt the overall GQ structure of the native thrombin-binding aptamer. 

### 2.2. 5FurU Position Influences TBA Structural Dynamics and Intramolecular Interactions, Which Impact TBA Stability and Photophysical Properties

**Figure 3:**
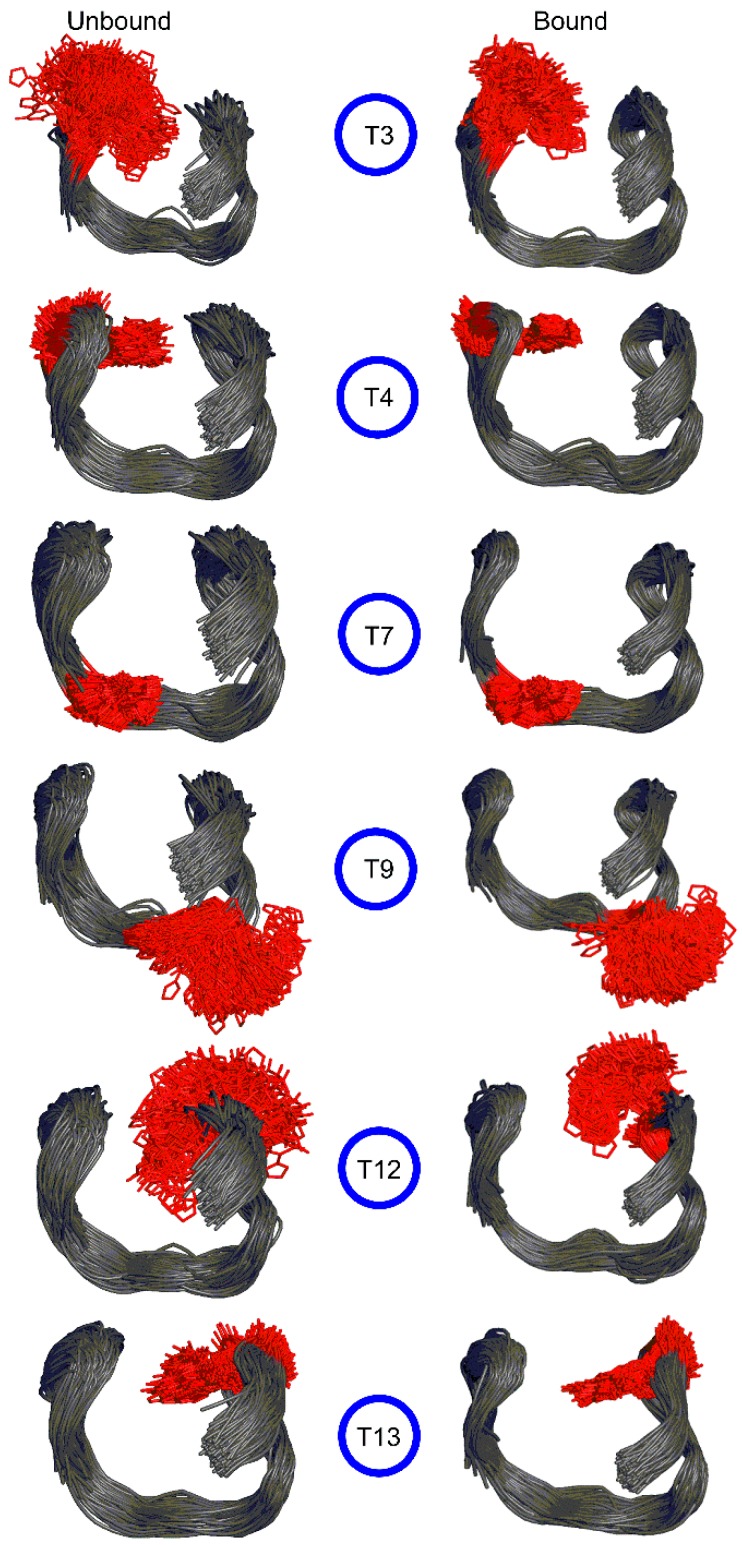
MD structures taken at 1 ns intervals of the unbound (left) and thrombin bound (right) TBA containing 5FurU at various positions (red) overlaid with respect to the aptamer backbone.

Similar to the T positions in the native TBA, 5FurU adopted the *anti* glycosidic conformation at all modification sites, with the average χ dihedral angle (∠(O4′C1′N1C2)) falling between ~195° and 240° (Figure S4). The 5-furyl moiety and nucleobase remained coplanar throughout the simulation regardless of the modification position, with the average θ (∠(C6C5C7O8)) being ~ 0 ± 7° (Figures S5 and S6). Nevertheless, the location of the modified base with respect to the surrounding nucleobases was dynamic (Figure 3, left), with the degree of flexibility depending on the modification position. Key dihedral angles in the aptamer backbone suggested that the change in the 5FurU orientation in the aptamer was primarily due to deviations in the δ dihedral angle (∠(C5′C4′C3′O3′), Figure S7), as well as the χ glycosidic dihedral angle (Figure S4). The distribution of these two dihedral angles was greater for 5FurU at positions 3, 9 and 12 than the unmodified T at the same position (Figure S8). These widespread 5FurU conformations correlated with the base being solvent-exposed and lacking strong intramolecular interactions at these positions (Figure 2; Table 2 and Table S6). In contrast, although more flexible than native T at the same position, the modified base conformations were more restricted at positions 4, 7, and 13 (Figure 3 and Figure S8). This likely arose due to stable (>7 kcal/mol) stacking interactions between the modified base at positions 4, 7 or 13 and the adjacent bases. In particular, 5FurU strongly stacked with G2 and G5 at position 4, with G6 at position 7, and with G11 at position 13 (Table S6). 

Since the adopted conformations of the natural base at T3 and T12 were not as widely distributed as 5FurU at the same positions, it can be concluded that the 5-furyl thymine modification at these positions incited some degree of flexibility into the aptamer. Furthermore, our simulations reveal that the flexibility of nucleotides outside the modification site could also be affected (Figure S3). This highlights the impact of the modification on the structural dynamics of TBA. These calculated structural features of 5FurU–TBA directly correlate with the experimentally-observed relative stability of the native and modified TBA [28]. Specifically, thermal melting experiments [28] suggested that 5FurU incorporation at positions 3 and 12 decrease the stability of the aptamer, which our simulations suggested was likely due to increased aptamer dynamics and solvent accessibility of the modification site. 

**Figure 4:**
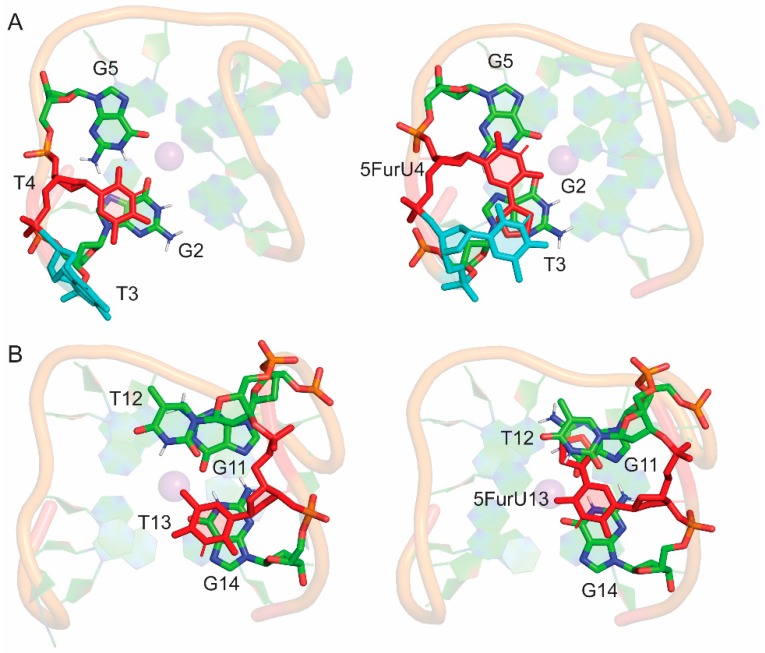
(**A**) Stacking interactions of the T4 base in the native (left) and modified (right) TBA. (**B**) Stacking interactions of the T13 base in the native (left) and modified (right) TBA.

In contrast to positions 3 and 12, the modifications at positions 4 and 13 imparted local changes in the aptamer structure that adjusted the relative orientations of adjacent nucleobases and improved nucleobase–nucleobase stacking interactions. Specifically, 5FurU at position 4 rearranged the backbone conformation of the adjacent T3 nucleotide, such that 5FurU was sandwiched between T3 and G2, as well as G5 (Figure 4). Indeed, the G2–T4, T3–T4 and T4–G5 stacking interactions each increased by up to ~5 kcal/mol upon T4 modification (Table S6). Furthermore, although a T13 modification did not significantly change the magnitude of stacking with G14, T12 repositioned due to the presence of the 5-furyl group such that the 5-furyl moiety was sandwiched between T12 and G11 (Figure 4). As a result, the G11–T13 and T12–T13 stacking interactions each became more stable by up to ~4 kcal/mol (Table S6). Our predicted enhanced stacking of the bases at positions 4 and 13 upon modification correlate with the experimentally-reported increased aptamer stability upon 5FurU incorporation at either site [28].

In addition to differential stability, the position of 5FurU in TBA has been shown to influence the photophysical parameters of the aptamer [28]. Specifically, the relative emission intensity of 5FurU was high at positions 3 and 12, whereas 5FurU at positions 4 and 13 exhibited a quenched response, which was proposed to arise due to changes in polarity. We note that the modification site was highly dynamic and the probe was fully solvent exposed at positions 3 and 12 (Figure 3 and Table 2). In contrast, the 5-furyl moiety of the modified base was stacked between a G-tetrad and neighboring loop T at positions 4 and 13 (Figure 4), which reduced the flexibility of the modification site (Figure 3), sequestered the modified base from water (Table 2), and ultimately quenched the probe fluorescence emission. Hence, the differential emission intensity of the unbound modified TBA can be clearly attributed to changes in the probe environment.

Beyond the TT loops, experimental studies have revealed that the incorporation of 5FurU at T7 leads to an enhanced fluorescence intensity and a decreased aptamer stability, while modification at T9 increases aptamer stability and only slightly improves fluorescence emission [28]. This was interpreted to imply that 5FurU is solvent-exposed at T7 and stacked with neighboring bases at T9. Though this proposal is consistent with the trends in our MD structural and published biophysical data for modifications to the TT loop positions [28], it contrasts with the global conformation of the T7 and T9 modified TBAs predicted in the present work, in which 5FurU predominantly stacked with G8 at T7, and was highly flexible and solvent-exposed at T9 (Figure 3 and Figure S9). Nevertheless, our predicted modified TBA structures were consistent with available crystal structures of the native [11] and other modified TBAs [21]. Furthermore, we note that 5FurU at position T9 was highly dynamic (Figure 3 and Figure S9). Indeed, conformations in which 5FurU at T9 stacked with G8 occurred for ~54% of the simulation trajectory, which would increase aptamer stability but quench the fluorescence response. Meanwhile, the probe was solvent-exposed in the remaining conformations, which rationalizes a slight increase in fluorescence intensity [28]. Regardless, we note that different orientations of the modified T7 and T9 loop residues may arise upon the folding of the modified aptamer. Due to the uncertainties surrounding the probe conformation in the TGT loop positions and the fact that the T7 and T9 modified TBA showed little-to-no change in fluorescence response upon thrombin binding [28], the modifications at T7 and T9 will not be further discussed.

### 2.3. 5FurU Modification Does Not Affect the Overall Conformation of TBA Bound to Thrombin

To understand the impact of chemical modifications on TBA–thrombin binding, we performed 0.5 μs MD simulations on the native and six modified TBA–thrombin complexes. All models were built from an X-ray crystal structure of a human α-thrombin–aptamer complex (PDB ID: 4DII), which suggested that the molecular recognition of the 15-mer TBA by thrombin involves the two TT loops [11]. The overall crystal structure geometry of the unmodified TBA–thrombin complex was maintained over the course of the MD simulation (Figures S10 and S11; Table S1). Specifically, the two G-tetrads were conserved (Table S7), with Hoogsteen–edge interactions between neighboring guanines mediated by two strong N1-H···O6 and N2-H···N7 hydrogen bonds (Tables S8 and S9). The K^+^ ion was positioned between the two G-tetrads and coordinated with the O6 atoms of the G-bases, with an average distance of ~2.7–2.8 Å (Table S10). 

In the native TBA–thrombin complex, nucleobases in the TT loops were located at the DNA–protein interface. As a result, the dynamics of the loop nucleobases were less in the aptamer–protein complex compared to the unbound TBA (Figure S12), especially at T3, T12, and T13. A total of 496 atomic contacts occurred between the nucleobase and amino acid residues at the DNA–protein interface over the MD simulation. Key contacts included interactions between T3 and Ile36, His93, Glu99, Ile102 and Tyr141; T4 with Arg97, Arg100 and Asn101; T12 with Arg89 and Tyr98; and T13 with Arg97, Tyr98 and Arg100 (Figure S13). Other than the TT loop bases, G2 and G14 interacted with Arg97, while G5 and G11 interacted with Arg100, albeit to a lesser extent compared to the TT loop bases. These nucleobase–amino acid contacts provided stability to the TBA–thrombin complex, with some of the interactions being particularly strong (up to –22 kcal/mol, Table S11). The TGT nucleobases were located far from the thrombin binding site (exosite–I) and did not interact with the protein (Figure S10). In addition to nucleobase–amino acid interactions, improved stacking interactions between the G-tetrad and the T-rich loops within TBA occurred upon protein binding, which further stabilized the TBA–thrombin complex. Specifically, the G2–T4 and G11–T13 stacking interactions were ~4 kcal/mol more stable in the presence of thrombin (Table S12). This was consistent with experimental data, reflecting an increase in the thermal stability of the G-quadruplex upon thrombin binding [11]. 

**Figure 5:**
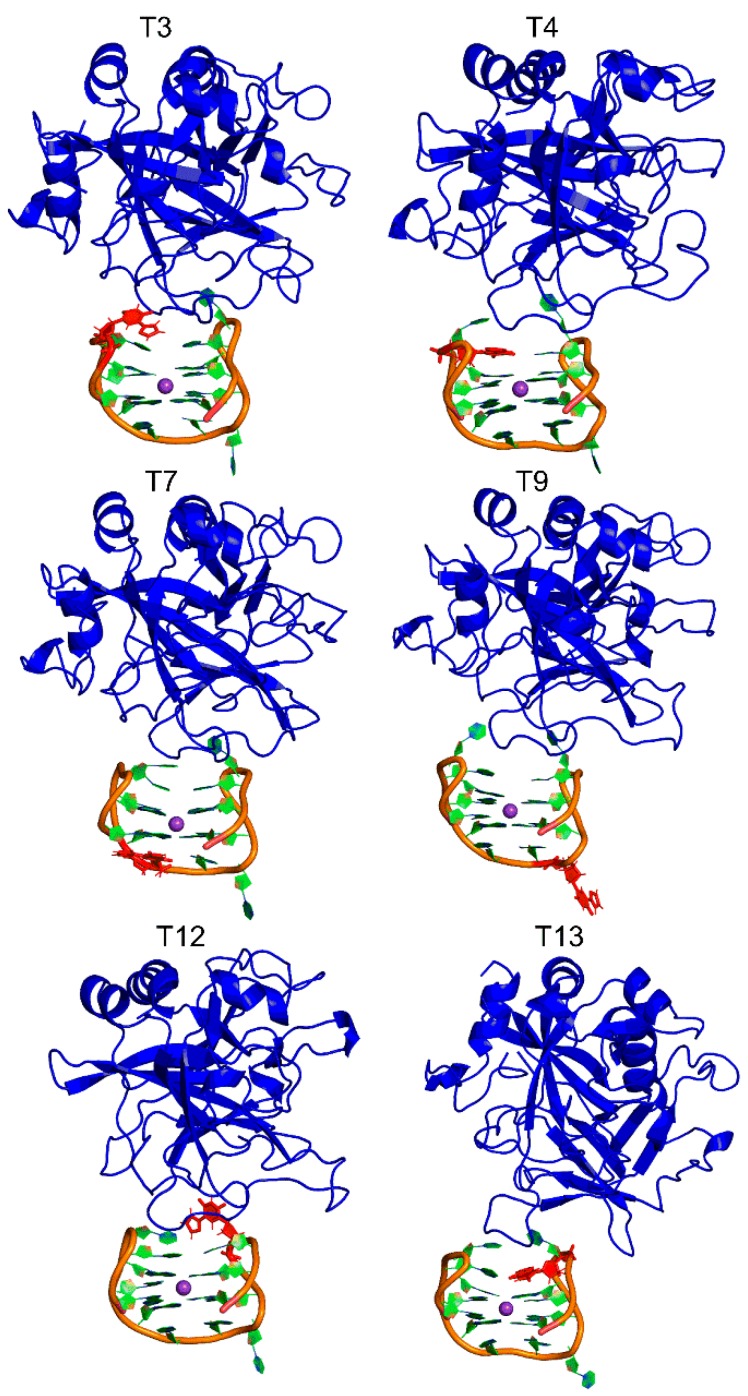
Representative MD structures of TBA containing 5FurU (red) bound to the thrombin protein (blue).

The representative MD structures for each modified TBA–thrombin complex highlighted that 5FurU does not significantly impact the overall structure of the complex, regardless of location (Figure 5). Indeed, the backbone RMSD was similar for the native and modified TBA–thrombin complexes (Table S1 and Figure S11). Likewise, G-quadruplex folding was conserved in the modified TBA–thrombin aptamer, where the G-tetrads were mediated by strong hydrogen bonds (Tables S7–S9), and the G-tetrad bases were well coordinated with the K^+^ ion (Table S10). As discussed for native TBA, the modified TBA exhibited less structural deviations in the backbone, as well as several nucleotides (including the TT loop regions), when complexed to thrombin (Table S1 and Figure S12). Together, this suggests that the modified TBA was still bound to thrombin with a structure comparable to the native TBA–thrombin complex. Indeed, although the native TBA–thrombin complex had a greater number of nucleobase–amino acid contacts than its modified counterparts, over 78% of contacts were preserved at the DNA–protein interface upon T modification. Nevertheless, discrete nucleobase–amino acid interactions at the TT loop positions were impacted by the incorporation of 5FurU, which has direct consequences for binding affinity and probe response.

### 2.4. 5FurU Position Differentially Affects Interactions at the TBA–Thrombin Interface, which Rationalizes Aptamer Binding Affinity and Probe Response

5FurU in the TBA–thrombin aptamer adopted the *anti* glycosidic conformation similar to the bound native and unbound modified aptamers (Figures S14 and S15). There was no significant deviation in modification–nucleobase linker θ dihedral angle (∠(C6C5C7O8)), which placed the 5-furyl moiety and the nucleobase in a coplanar arrangement throughout the MD simulation, regardless of the modification position (Figures S16 and S17). Thus, the differential emission intensity of the TBA–thrombin complex likely arose due to changes in the overall structural dynamics, solvent-exposure and/or protein environment of the probe.

**Figure 6:**
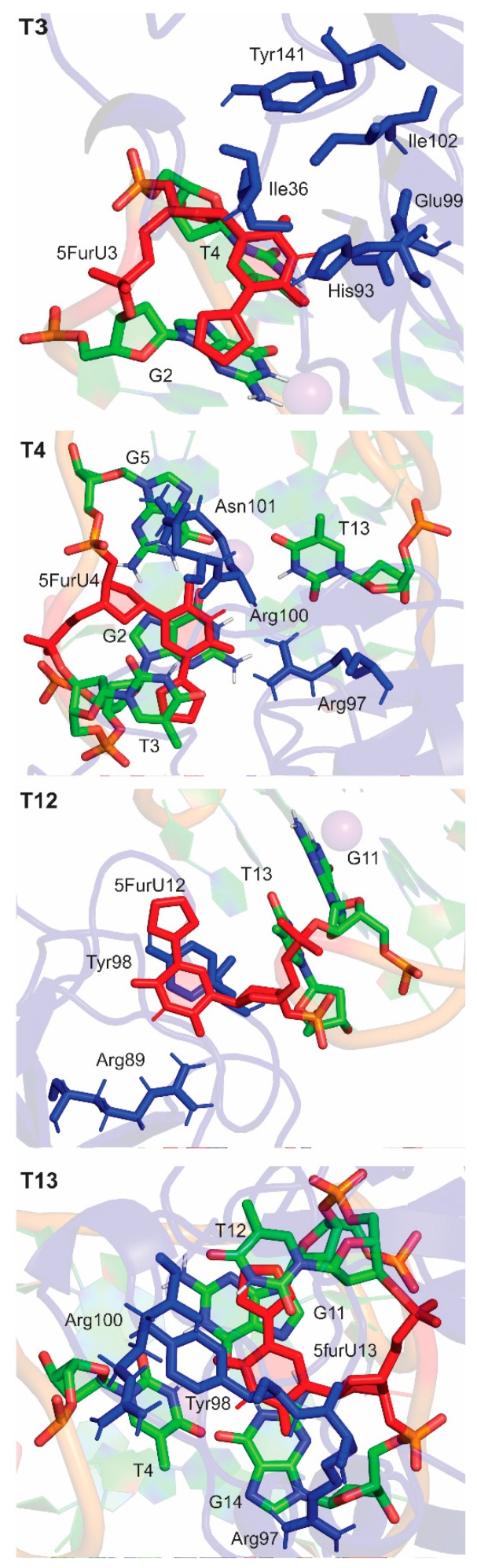
Nucleobase (green) and amino acid (blue) residues surrounding 5FurU at the T3, T4, T12 or T13 positions (red) at the DNA–protein interface in the modified TBA–thrombin complexes.

Protein binding changed the orientation of some nucleobases in the modified TBA at the DNA–protein interface without affecting the global conformation of TBA (Figure 6). This rearrangement significantly altered the internal nucleobase–nucleobase stacking interactions for the TBA-modified positions 4 and 13, where the 5-furyl moiety was sandwiched between surrounding bases in the unbound aptamer (Tables S6 and S12). Specifically, the stacking interaction between 5FurU at position 4 and G5 decreased in strength by ~ 6 kcal/mol. In contrast, at position 13, T12 stacked more strongly with 5FurU upon binding to thrombin (by ~ 3 kcal/mol). This indicates that the presence of the protein impacts the stability of TBA modified at positions 4 and 13, which correlates with the reported decreased binding affinity for these 5FurU-containing aptamers [28].

**Figure 7:**
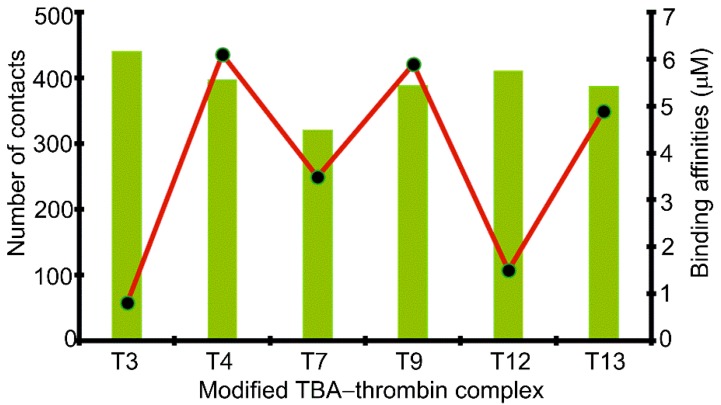
Total number of contacts at the DNA–protein interface in the modified TBA–thrombin complexes. Experimental binding affinities of the TBA–thrombin complexes are indicated by the black dots.

A comparison of the modified aptamers revealed that the 5FurU position greatly influenced the number and strength of the DNA–protein interactions (Figure 6 and Figure 7 and Table S11), and the associated trends nicely correlate with the experimental binding affinities [28]. For example, the T3 modified aptamer bound to the protein with the greatest number of contacts (Figure 7), and the magnitude of each interaction was significant (up to –23 kcal/mol), with several of the native TBA–thrombin interactions (including T3–Glu99) being strengthened upon the incorporation of 5FurU (Table S11). Similarly, the T12 modified aptamer had a large number of highly stable TBA–thrombin contacts and maintained key interactions with Arg89 and Tyr98. In contrast, TBA containing 5FurU at the T13 position had the smallest number of DNA–protein atomic contacts among all modified aptamers (Figure 7), and the majority of the interactions were much less stable than the corresponding native TBA–thrombin contact (by up to 15 kcal/mol, Table S11). Furthermore, 5FurU at T13 remained stacked with T12 upon target binding (Figure 4 and Figure 6), which prevented strong contacts between the protein and modified base. Similarly, 5FurU at the T4 position remained stacked with T3 upon binding to thrombin (Figure S6). These findings rationalize the reported greater binding affinity of the T3 and T12 modified TBA, as well as the weaker aptamer binding when 5FurU is located at T4 or T13 [28]. 

In the presence of thrombin, the flexibility of 5FurU was reduced for all modification positions (Figure 3). This mainly arose due to nucleobase–amino acid interactions at the DNA–protein interface, thus restricting fluctuations in the δ and χ backbone dihedral angles of the modified nucleobases (Figures S7, S8 and S18). Furthermore, the solvent accessibility of the modification decreased upon TBA–thrombin binding (Table 2). Nevertheless, at the T3 and T12 positions, 5FurU remained significantly solvent-exposed in the modified TBA–thrombin complex. This access to water coupled with the reduced flexibility at the modification site rationalizes the observed enhanced probe fluorescence upon target binding [28]. In contrast, although 5FurU flexibility at positions T4 and T13 was also reduced upon the formation of the TBA–thrombin complex, the modified nucleobase was sandwiched between surrounding nucleobase in both the bound and unbound structures, which would prohibit a probe response in the presence of the target. Thus, our data provide structural insights into the experimental observations that 5FurU properties can be affected by the local environment, and they explain the two-fold increase in the probe emission intensity upon thrombin addition for modification at positions 3 and 12 but the lack of response for 5FurU incorporation at positions 4 and 13 [28]. 

## 3. Methods

A 2.05 Å X-ray crystal structure corresponding to the human α-thrombin–aptamer complex with K^+^ bound to the G-quadruplex (PDB ID: 4DII) was used to build initial structures for MD simulations. In this structure, 15-mer TBA (5′–GGTTGGTGTGGTTGG–3′) is bound at exosite-I of the thrombin protein. Unresolved residues (Trp146–Gly155) were manually added using GaussView 6.0 [56]. The initial structure of unbound TBA was obtained by extracting the DNA coordinates from the same crystal structure of the α-thrombin–aptamer complex. Modified aptamers were built by replacing the C5 methyl group of the T base at positions 3, 4, 7, 9, 12, or 13 (Table 1) with the 5-furyl moiety using GaussView 6.0. The initial modified nucleoside conformation was adjusted by visual inspection to maintain the *anti* glycosidic conformation while minimizing steric clashes with neighboring nucleobases. 

The parmbsc1 force field [57] was used to simulate natural DNA nucleotides, while AMBER param14SB parameters were used for proteins. The parameters for the 5FurU modified base were supplemented by a generalized amber force field (GAFF) [58], with partial atomic charges calculated with RED.v.III.4 using the RESP-A1 scheme [59]. All amino acids and nucleotides were assigned physiological protonation states using the tLEAP module of AMBER 16 [60]. Each aptamer system was neutralized with Na^+^ ions (13 for TBA and 8 for TBA–thrombin) and solvated in an explicit TIP3P octahedral water box, ensuring that the edge of the box was at least 10.0 Å from the edge of the solute in each direction. 

The solvent molecules and ions were minimized with 500 steps of steepest descent and 500 steps of conjugate gradient minimization using a nonbonded cutoff of 10 Å, while the protein and TBA were constrained using a 500.0 kcal mol^−1^ Å^−2^ force constant. The entire system was then minimized using 1000 steps of unrestrained steepest descent, followed by 1500 steps of unrestrained conjugate gradient minimization. Subsequently, the system was heated from 0 to 300 K during a 100 ps solute-restrained (10 kcal mol^−1^ Å^−2^) equilibration phase, using a 1 fs time step. This was followed by a 20 ns (unconstrained) trial MD simulation under NPT conditions (1 atm and 300 K) with a time step of 2 fs and SHAKE. The periodic boundary condition was employed for all steps. Each production simulation was subsequently run for 0.5 μs starting from conformations obtained from the last frame of the trial simulations using the PMEMD module of AMBER 16 [60]. Thus, a total of 7.0 μs production simulations were performed for the 14 models, including one native TBA, six modified TBAs, one native TBA–thrombin complex, and six modified TBA–thrombin complexes.

Trajectory analysis was completed using the CPPTRAJ module [61] of AMBER 16. The root-mean-square deviations (RMSD) in the protein and TBA backbones over the production phase were analyzed for each simulation to ensure that the system was stable (Table S1). Each trajectory was sampled every 20 ps over the course of the production phase. For all modified aptamers and aptamer–thrombin complexes, clustering was carried out with respect to the configuration of the modified base, and the reported MD representative structure was obtained from the dominant cluster, which had an occupancy >90% over the simulation trajectory in all cases. All reported average backbone RMSD, per-residue RMSD, backbone dihedral angles, χ (∠(O4′C1′N1C2)) and θ (∠(C6C5C7O8)) torsional angles, solvent accessible surface area (SASA), and hydrogen-bonding parameters were calculated over the entire MD simulation trajectory. To determine hydrogen-bonding occupancies, 120° angle and 3.4 Å distance cut-offs were imposed. In the TBA–thrombin complexes, the number of intermolecular contacts at the protein–DNA interface were determined over the MD simulation by evaluating the number of amino acid and nucleobase atoms within 5 Å with reference to the initial structure. Furthermore, the magnitude of the nucleobase–nucleobase and nucleobase–amino acid interactions were calculated by extracting the atomic coordinates from the simulation trajectory at 5 ns intervals. The deoxyribose and Cα of the amino acids were replaced with hydrogen atoms. Hydrogen-only optimizations were performed using B3LYP/6-31+G(d,p), and the noncovalent interaction energies were calculated using B3LYP-D3(BJ)/6-311+G(2df,p). All B3LYP calculations were performed using Gaussian 16 [62].

## 4. Conclusions

The present work highlights the impact of the fluorescent 5FurU base within the three unique loops of TBA on the unbound aptamer and TBA–thrombin complex. 5FurU is an excellent T-base analog, as the probe maintains the overall GQ structure of TBA in the presence and absence of the target protein. The 5FurU modification position impacts the structural dynamics of the probe, changes the solvent exposure of 5FurU, and fine tunes interactions within the aptamer and at the TBA–thrombin interface. Furthermore, the measured changes directly correlate with reported differential biophysical properties of the aptamer, the binding affinity of modified TBA to thrombin, and the probe response in the presence of thrombin [28]. Specifically, 5FurU at positions 3 and 12 in unbound TBA is highly dynamic and solvent-exposed, which destabilizes the aptamer while affording a strong fluorescence emission. In contrast, 5FurU at positions 4 and 13 is quenched because the 5-furyl moiety is sandwiched between neighboring nucleobases. When modified TBA is bound to thrombin, 5FurU at positions 3 and 12 maintains solvent exposure, but is less dynamic, which results in an enhanced emission intensity in the presence of the target. However, there is a lack of probe response at positions 4 and 13 upon thrombin binding, since 5FurU is surrounded by neighboring nucleobases in both the unbound and bound aptamer. Overall, this study highlights the ability to use the computational protocols implement in the present work in a predictive way to understand the probe environment and aid the design of future modifications for aptamer recognition. Our data also further underscore that TBA positions 3 and 12 are promising modification sites for fluorescent probes and should be further explored to fine tune the probe design for aptamer recognition for use as biosensors and/or therapeutics. 

## Figures and Tables

**Table 1:** TBA variant sequences considered in the present work.

TBA	Sequence ^a^
Native	5′–GGTTGGTGTGGTTGG–3′
T3	5′–GGXTGGTGTGGTTGG–3′
T4	5′–GGTXGGTGTGGTTGG–3′
T7	5′–GGTTGGXGTGGTTGG–3′
T9	5′–GGTTGGTGXGGTTGG–3′
T12	5′–GGTTGGTGTGGXTGG–3′
T13	5′–GGTTGGTGTGGTXGG–3′

^a^ X = 5FurU modification site.

**Table 2:** Average (and standard deviation in parentheses) solvent accessible surface area (SASA, Å^2^) of native or modified T in each TBA position throughout 0.5 μs molecular dynamics (MD) simulations.

Position	Unbound			Bound		
	Native	Modified	Δ^a^	Native	Modified	Δ^a^
T3	266.5	314.8	48.4	194.2	182.5	−11.7
	(20.7)	(19.6)		(34.9)	(27.1)	
T4	167.7	151.6	−16.2	126.9	129.0	2.1
	(26.6)	(17.3)		(18.5)	(12.9)	
T7	230.8	261.3	30.5	233.3	263.9	30.6
	(7.1)	(8.4)		(6.8)	(8.1)	
T9	290.8	293.4	2.6	290.7	332.8	42.1
	(6.8)	(36.9)		(7.2)	(11.2)	
T12	236.6	313.2	76.6	207.5	265.1	57.6
	(32.5)	(20.7)		(23.0)	(21.0)	
T13	171.1	147.5	−23.6	80.8	76.6	−4.2
	(22.3)	(18.2)		(17.1)	(15.6)	

^a^ SASA of the modified 5FurU base minus the SASA of the native T-base at the same position.

## Supplementary Materials

The following are available online. Table S1. Average backbone RMSD for each TBA and TBA–thrombin complexes throughout 0.5 μs MD simulations; Table S2. Hydrogen–bonding occupancies in the G–tetrads for unbound native and modified TBA throughout 0.5 μs MD simulations; Table S3. Average distances between hydrogen-bond donor and acceptor atoms in G–tetrads for unbound native and modified TBA throughout 0.5 μs MD simulations; Table S4. Average hydrogen-bond angle in G–tetrads for unbound native and modified TBA throughout 0.5 μs MD simulations; Table S5. Average K^+^ coordination distances in unbound native and modified TBA throughout 0.5 μs MD simulations; Table S6. Average nucleobase stacking interactions in unbound native and modified TBA throughout 0.5 μs MD simulations; Table S7. Hydrogen–bonding occupancies in the G–tetrads in bound native and modified TBA–thrombin throughout 0.5 μs MD simulations; Table S8. Average distances of hydrogen-bond donor and acceptor atoms in G–tetrads for bound native and modified TBA–thrombin complexes throughout 0.5 μs MD simulations; Table S9. Average hydrogen-bond angle in G–tetrads for bound native and modified TBA–thrombin complexes throughout 0.5 μs MD simulations; Table S10. Average K^+^ coordination distances in bound native and modified TBA–thrombin complexes throughout 0.5 μs MD simulations; Table S11. Average nucleobase–amino acid interactions in the native and modified TBA–thrombin complexes throughout 0.5 μs MD simulations; Table S12. Average nucleobase stacking interactions in the bound native and modified TBA–thrombin complexes throughout 0.5 μs MD simulations; Figure S1. (A) Schematic representation of TBA, with modification sites highlighted in red. (B) MD representative structure of native TBA. (C) Overlay of DNA aptamer from crystal and MD representative structures of unbound native TBA; Figure S2. Structural deviations in the backbone with respect to the first simulation frame for each modified TBA compared to native TBA throughout 0.5 μs MD simulations; Figure S3. Average structural deviation in each nucleotide with respect to the first simulation frame for each modified TBA compared to native TBA throughout 0.5 μs MD simulations; Figure S4. Probability distribution in the χ torsion angle for the base at position T3, T4, T7, T9, T12 or T13 in native and modified TBA; Figure S5. Overlay of the modified 5FurU nucleobase with respect to nucleobase ring for different modified TBA; Figure S6. Probability distribution in the θ torsion angle of 5FurU for each modified TBA calculated over the 0.5 μs MD simulations; Figure S7. (A) Definition of nucleotide backbone torsion angles. (B) Probability distribution in the nucleotide backbone torsion angles in the modified 5FurU base at each TBA position; Figure S8. Scatter plot of the χ versus δ torsion angles for the native and modified base at various positions in unbound and bound TBA; Figure S9. Stacking interactions of the T7 and T9 bases in unbound native and modified TBA aptamer; Figure S10. (A) MD representative structure of the native TBA–thrombin complex. (B) Overlay of crystal and MD representative structures of TBA–thrombin complex; Figure S11. Structural deviations in the backbone with respect to the first simulation frame for native and modified TBA–thrombin complexes throughout 0.5 μs MD simulations; Figure S12. Average structural deviations with respect to the first simulation frame for each nucleotide in native and modified unbound TBA compared to the corresponding TBA–thrombin complex throughout the 0.5 μs MD simulations; Figure S13. Nucleobase and amino acid residues surrounding the T base at the T3, T4, T12 or T13 position at the DNA–protein interface in native TBA–thrombin complexes; Figure S14. Probability distribution in the χ torsion angle for the modified base at position T3, T4, T7, T9, T12 or T13 in the modified TBA–thrombin complex compared to the native TBA–thrombin complex calculated over the 0.5 μs MD simulations; Figure S15. Probability distribution in the χ torsion angle for the modified base at position T3, T4, T7, T9, T12 or T13 in TBA and the TBA–thrombin complex calculated over the 0.5 μs MD simulation; Figure S16. Overlay of the modified 5FurU nucleobase with respect to nucleobase ring for different modified TBA–thrombin complexes; Figure S17. Probability distribution in the θ torsion angle for each modified TBA–thrombin complex calculated over the 0.5 μs MD simulations; Figure S18. (A) Definition of nucleotide backbone torsion angles. (B) Probability distribution in the nucleotide backbone torsion angles in the modified 5FurU base at each position in TBA–thrombin complexes.
