# Supplementary material for: Impact of the Position of the Chemically Modified 5-Furyl-2′-Deoxyuridine Nucleoside on the Thrombin DNA Aptamer–Protein Complex: Structural Insights into Aptamer Response from MD Simulations
Source: Molecules. 2019 Aug 10;24(16):2908. doi: 10.3390/molecules24162908 (PMC6720718; doi:10.3390/molecules24162908)
Supplement: Supplementary file 1 [file molecules-24-02908-s001.pdf]

Supporting Material for

Impact of the position of the chemically modified 5–furyl–2′–deoxyuridine nucleoside on the thrombin DNA aptamer–protein complex: Structural insights into aptamer response from MD simulations

Preethi Seelam Prabhakar,[1] Richard A. Manderville[2] and Stacey D. Wetmore[1]*

[1]*Department of Chemistry and Biochemistry, University of Lethbridge, 4401 University Drive West, Lethbridge, Alberta, Canada T1K 3M4*

[2]*Department of Chemistry and Toxicology, University of Guelph, Guelph, Ontario, Canada N1G 2W1*

* Correspondance : stacey.wetmore@uleth.ca. Tel.: (403) 329–2323. ORCID: 0000–0002–5801–3942

Table of Contents

Table S1. Average backbone RMSD (standard deviation in parentheses, Å) for each TBA and TBA–thrombin complexes throughout 0.5 μs MD simulations……………………………………5

Table S2. Hydrogen–bonding occupancies (%) in the G–tetrads for unbound native and modified TBA throughout 0.5 μs MD simulations……………………………………………………...6

Table S3. Average distances (Å) between hydrogen-bond donor and acceptor atoms in G–tetrads for unbound native and modified TBA throughout 0.5 μs MD simulations……………………….7

Table S4. Average hydrogen-bond angle (°) in G–tetrads for unbound native and modified TBA throughout 0.5 μs MD simulations…………………………………………………………...8

Table S5. Average K^+ coordination distances (standard deviation in parentheses, Å) in unbound native and modified TBA throughout 0.5 μs MD simulations…………………………….……9

Table S6. Average nucleobase stacking interactions (standard deviation in parentheses, kcal/mol) in unbound native and modified TBA throughout 0.5 μs MD simulations………………………10

Table S7. Hydrogen–bonding occupancies (%) in the G–tetrads in bound native and modified TBA–thrombin throughout 0.5 μs MD simulations………………………………………………11

Table S8. Average distances (Å) of hydrogen-bond donor and acceptor atoms in G–tetrads for bound native and modified TBA–thrombin complexes throughout 0.5 μs MD simulations……...12

Table S9. Average hydrogen-bond angle (°) in G–tetrads for bound native and modified TBA–thrombin complexes throughout 0.5 μs MD simulations…………………………………………13

Table S10. Average K^+ coordination distances (standard deviation in parentheses, Å) in bound native and modified TBA–thrombin complexes throughout 0.5 μs MD simulations……………..14

Table S11. Average nucleobase–amino acid interactions (standard deviation in parentheses, kcal/mol) in the native and modified TBA–thrombin complexes throughout 0.5 μs MD simulations……………………………………………………………………………….....15

Table S12. Average nucleobase stacking interactions (standard deviation in parentheses, kcal/mol) in the bound native and modified TBA–thrombin complexes throughout 0.5 μs MD simulations……………………………………………………………………………….....16

Figure S1. (A) Schematic representation of TBA, with modification sites highlighted in red. (B) MD representative structure of native TBA. (C) Overlay of DNA aptamer from crystal (PDB ID: 4DII, red) and MD representative (blue) structures of unbound native TBA…………………...17

Figure S2. Structural deviations in the backbone (RMSD, Å) with respect to the first simulation frame for each modified TBA compared to native TBA (dark green) throughout 0.5 μs MD simulations……………………………………………………………………………….....18

Figure S3. Average structural deviation in each nucleotide (RMSD, Å) with respect to the first simulation frame for each modified TBA compared to native TBA (dark green) throughout 0.5 μs MD simulations……………………………………………………………………………..19
Figure S4. Probability distribution in the χ torsion angle (∠(O4′C1′N1C2), degrees) for the base at position T3, T4, T7, T9, T12 or T13 in native (dark green) and modified TBA………………...20

Figure S5. Overlay of the modified 5FurU nucleobase with respect to nucleobase ring for different modified TBA…………………………………………………………………………........21

Figure S6. Probability distribution in the θ torsion angle of 5FurU (∠(C6C5C7O8), degrees) for each modified TBA calculated over the 0.5 μs MD simulations……………………………......22

Figure S7. (A) Definition of nucleotide backbone torsion angles (α, β, γ, δ, ε, and ζ). (B) Probability distribution in the nucleotide backbone torsion angles in the modified 5FurU base at each TBA position……………………………………………………………………………………...23

Figure S8. Scatter plot of the χ (∠(O4′C1′N1C2)) versus δ (∠(C5′C4′C3′O3′)) torsion angles for the native (dark green) and modified base at various positions in unbound (left) and bound (right) TBA………………………………………………………………………………..…..24

Figure S9. Stacking interactions of the T7 (top, red) and T9 (bottom, red) bases in unbound native (left) and modified (middle and right) TBA aptamer……………………………………….25

Figure S10. (A) MD representative structure of the native TBA–thrombin complex. (B) Overlay of crystal (PDB ID: 4DII, red) and MD representative (blue) structures of TBA–thrombin complex……………………………………………………………………………………..26

Figure S11. Structural deviations in the backbone (RMSD, Å) with respect to the first simulation frame for native (dark green) and modified TBA–thrombin complexes throughout 0.5 μs MD simulations………………………………………………………………………...…..27

Figure S12. Average structural deviations (RMSD, Å) with respect to the first simulation frame for each nucleotide in native and modified unbound TBA (black circles) compared to the corresponding TBA–thrombin complex (colored circles) throughout the 0.5 μs MD simulation……………………………………………………………………………………...28

Figure S13. Nucleobase (green) and amino acid (blue) residues surrounding the native T base at the T3, T4, T12 or T13 position (red) at the DNA–protein interface in native TBA–thrombin complexes…………………………………………………………………………....…29

Figure S14. Probability distribution in the χ torsion angle (∠(O4′C1′N1C2), degrees) for the modified base at position T3, T4, T7, T9, T12 or T13 in the modified TBA–thrombin complex compared to the native TBA–thrombin complex (dark green) calculated over the 0.5 μs MD simulations……………………………………………………………………………….....30

Figure S15. Probability distribution in the χ torsion angle (∠(O4′C1′N1C2), degrees) for the modified base at position T3, T4, T7, T9, T12 or T13 in TBA (dotted line) and the TBA–thrombin complex (line) calculated over the 0.5 μs MD simulation……………………………………..31

Figure S16. Overlay of the modified 5FurU nucleobase with respect to nucleobase ring for different modified TBA–thrombin complexes…………………………………………………...32

Figure S17. Probability distribution in the θ torsion angle (∠(C6C5C7O8), degrees) for each modified TBA–thrombin complex calculated over the 0.5 μs MD simulations………………..…33

Figure S18. (A) Definition of nucleotide backbone torsion angles (α, β, γ, δ, ε, and ζ). (B) Probability distribution in the nucleotide backbone torsion angles in the modified 5FurU base at each position in TBA–thrombin complexes……………………………………………………...34

Table S1. Average backbone RMSD (standard deviation in parentheses, Å) for each TBA and TBA–thrombin complexes throughout 0.5 μs MD simulations.[a]

	TBA	TBA–thrombin	
	DNA	DNA	DNA + Protein
	Avg. RMSD	Avg. RMSD	Avg. RMSD
Native	1.194 (0.224)	1.057 (0.135)	1.748 (0.252)
T3	1.011 (0.213)	1.265 (0.180)	1.569 (0.250)
T4	1.183 (0.221)	1.014 (0.172)	1.617 (0.171)
T7	1.230 (0.268)	0.822 (0.149)	1.736 (0.181)
T9	1.507 (0.287)	1.163 (0.130)	1.842 (0.382)
T12	1.238 (0.260)	1.002 (0.296)	1.491 (0.318)
T13	1.268 (0.211)	1.190 (0.243)	3.371 (0.720)

[a] RMSD is calculated with respect to the first simulation frame.

Table S2. Hydrogen–bonding occupancies (%) in the G–tetrads for unbound native and modified TBA throughout 0.5 μs MD simulations.[a]

Nucleobase Interactions	H–Bond Interactions	H–bond Occupancies (%)						
		Native	T3	T4	T7	T9	T12	T13
G1 – G6	N1–H···O6	99	100	99	100	100	100	100
	N2–H···N7	100	99	100	100	97	100	100
G1 – G15	N7···H–N2	99	99	99	100	99	99	99
	O6···H–N1	99	100	100	100	100	99	100
G2 – G5	N7···H–N2	100	100	100	100	100	100	100
	O6···H–N1	100	100	100	100	100	100	100
G2 – G14	N1–H···O6	100	100	100	100	100	100	100
	N2–H···N7	99	99	98	99	99	99	99
G5 – G11	N7···H–N2	99	99	99	99	100	100	99
	O6···H–N1	100	100	100	100	99	99	100
G6 – G10	N1–H···O6	100	100	100	100	99	99	100
	N2–H···N7	100	100	100	100	99	100	100
G10 – G15	N1–H···O6	100	100	100	100	100	100	100
	N2–H···N7	99	99	99	99	96	99	100
G11 – G14	N7···H–N2	100	100	100	100	100	100	100
	O6···H–N1	100	100	100	100	100	100	100

[a] Average values are calculated over the entire production MD simulation using 120° and 3.4 Å distance cut-off.

Table S3. Average distances (Å) between hydrogen-bond donor and acceptor atoms in G–tetrads for unbound native and modified TBA throughout 0.5 μs MD simulations.[a]

Nucleobase Interactions	H–Bond Interactions	Avg. Distances (Å)						
		Native	T3	T4	T7	T9	T12	T13
G1 – G6	N1–H···O6	3.0	2.9	2.9	2.9	2.9	2.9	2.9
	N2–H···N7	3.0	3.0	3.0	3.0	3.0	3.0	3.0
G1 – G15	N7···H–N2	3.0	3.0	3.0	3.0	3.0	3.0	3.0
	O6···H–N1	2.9	2.9	2.9	2.9	2.9	2.9	2.9
G2 – G5	N7···H–N2	2.9	3.0	2.9	3.0	3.0	3.0	3.0
	O6···H–N1	2.9	3.0	2.9	2.9	2.9	2.9	2.9
G2 – G14	N1–H···O6	3.0	2.9	2.9	2.9	2.9	2.9	2.9
	N2–H···N7	3.0	3.0	3.0	3.0	3.0	3.0	3.0
G5 – G11	N7···H–N2	3.0	3.0	3.0	3.0	3.0	3.0	3.0
	O6···H–N1	3.0	2.9	2.9	2.9	2.9	2.9	2.9
G6 – G10	N1–H···O6	2.9	2.9	2.9	2.9	2.9	2.9	2.9
	N2–H···N7	2.9	2.9	2.9	2.9	3.0	2.9	2.9
G10 – G15	N1–H···O6	2.9	2.9	2.9	2.9	2.9	2.9	2.9
	N2–H···N7	3.0	3.0	3.0	3.0	3.0	3.0	3.0
G11 – G14	N7···H–N2	3.0	2.9	2.9	2.9	2.9	2.9	2.9
	O6···H–N1	2.9	2.9	2.9	2.9	2.9	2.9	2.9

[a] Average values are calculated over the entire production MD simulation.

Table S4. Average hydrogen-bond angle (°) in G–tetrads for unbound native and modified TBA throughout 0.5 μs MD simulations.[a]

Nucleobase Interactions	H–Bond Interactions	Avg. ∠(D–H–A) Angle (°)						
		Native	T3	T4	T7	T9	T12	T13
G1 – G6	N1–H···O6	152	155	153	155	159	156	155
	N2–H···N7	162	161	162	162	156	161	162
G1 – G15	N7···H–N2	162	161	161	161	159	161	162
	O6···H–N1	157	158	158	157	160	158	157
G2 – G5	N7···H–N2	162	161	158	161	160	159	160
	O6···H–N1	155	158	160	158	158	159	159
G2 – G14	N1–H···O6	152	157	160	158	157	158	161
	N2–H···N7	164	161	159	161	161	161	160
G5 – G11	N7···H–N2	164	162	159	161	161	161	160
	O6···H–N1	155	157	160	158	157	158	160
G6 – G10	N1–H···O6	155	157	157	161	157	156	156
	N2–H···N7	163	161	161	157	158	162	161
G10 – G15	N1–H···O6	156	157	156	156	159	158	154
	N2–H···N7	163	162	163	162	157	161	164
G11 – G14	N7···H–N2	162	161	159	159	159	160	158
	O6···H–N1	156	159	161	160	159	159	161

[a] Average values are calculated over the entire production MD simulation.

Table S5. Average K^+ coordination distances (standard deviation in parentheses, Å) in unbound native and modified TBA throughout 0.5 μs MD simulations.[a]

	Native	T3	T4	T7	T9	T12	T13
K···G1(O6)	2.8 (0.2)	2.8 (0.1)	2.8 (0.2)	2.8 (0.2)	2.8 (0.1)	2.8 (0.2)	2.8 (0.2)
K···G2(O6)	2.7 (0.1)	2.8 (0.1)	2.8 (0.1)	2.8 (0.1)	2.8 (0.2)	2.8 (0.2)	2.8 (0.2)
K···G5(O6)	2.8 (0.1)	2.8 (0.2)	2.8 (0.2)	2.8 (0.1)	2.8 (0.2)	2.8 (0.2)	2.7 (0.1)
K···G6(O6)	2.8 (0.2)	2.8 (0.2)	2.8 (0.2)	2.8 (0.2)	2.9 (0.2)	2.8 (0.2)	2.8 (0.2)
K···G10(O6)	2.7 (0.1)	2.7 (0.1)	2.7 (0.1)	2.7 (0.1)	2.8 (0.1)	2.8 (0.1)	2.7 (0.1)
K···G11(O6)	2.8 (0.2)	2.8 (0.2)	2.8 (0.2)	2.8 (0.2)	2.8 (0.2)	2.8 (0.2)	2.8 (0.2)
K···G14(O6)	2.8 (0.1)	2.8 (0.1)	2.8 (0.1)	2.8 (0.1)	2.8 (0.2)	2.8 (0.2)	2.8 (0.1)
K···G15(O6)	2.8 (0.2)	2.8 (0.2)	2.9 (0.2)	2.8 (0.2)	2.8 (0.2)	2.8 (0.2)	2.8 (0.2)

[a] Average values are calculated over the entire production MD simulation.

Table S6. Average nucleobase stacking interactions (standard deviation in parentheses, kcal/mol) in unbound native and modified TBA throughout 0.5 μs MD simulations.[a]

	Native	T3	T4	T7	T9	T12	T13
G2–T4	–4.3 (2.7)	–6.1 (2.0)	–6.9 (2.1)	–6.0 (2.1)	–6.5 (2.2)	–5.3 (2.5)	–4.0 (3.4)
T3–T4	–0.9 (1.5)	–0.2 (0.4)	–4.6 (2.0)	–0.3 (1.2)	–0.1 (0.8)	–0.4 (1.1)	–1.3 (1.4)
T4–G5	–1.9 (0.7)	–2.0 (1.0)	–7.6 (1.2)	–2.0 (1.2)	–1.7 (1.2)	–3.0 (1.7)	–0.9 (1.6)
G6–T7	–7.3 (1.1)	–7.3 (1.5)	–7.3 (1.2)	–8.2 (1.3)	–5.4 (2.2)	–5.2 (2.8)	–7.4 (1.7)
G8–G10	–6.8 (1.2)	–7.1 (1.5)	–7.3 (1.2)	–7.3 (1.4)	–5.8 (1.7)	–6.8 (1.4)	–6.9 (1.3)
G11–T13	–3.6 (2.1)	–6.0 (2.1)	–7.1 (1.9)	–5.5 (2.5)	–5.6 (2.5)	–5.3 (2.2)	– 7.5 (2.3)
T13–G14	–1.6 (0.7)	–1.8 (1.1)	–1.8 (1.2)	–2.7 (1.3)	–2.5 (1.8)	–2.1 (1.5)	–1.8 (1.6)
T12–T13	–	–	–	–	–	–	–1.6 (1.6)

[a] Stacking interactions were calculated using B3LYP-D3(BJ)/6-311+G(2df,p) single-point calculations on structures taken at 5 ns intervals throughout the 0.5 μs MD simulation. See computational methods section in the main text for full details.

Table S7. Hydrogen–bonding occupancies (%) in the G–tetrads in bound native and modified TBA–thrombin throughout 0.5 μs MD simulations.[a]

Nucleobase Interactions	H–Bond Interactions	H–bond Occupancies (%)						
		Native	T3	T4	T7	T9	T12	T13
G1 – G6	N1–H···O6	99	99	99	99	99	99	99
	N2–H···N7	100	100	100	100	100	100	100
G1 – G15	N7···H–N2	99	99	100	99	100	99	99
	O6···H–N1	99	99	99	100	99	100	100
G2 – G5	N7···H–N2	100	100	100	100	100	100	100
	O6···H–N1	100	100	100	100	100	100	100
G2 – G14	N1–H···O6	99	99	99	100	99	100	100
	N2–H···N7	100	100	99	99	99	99	99
G5 – G11	N7···H–N2	100	100	100	100	100	100	99
	O6···H–N1	99	99	99	100	99	100	100
G6 – G10	N1–H···O6	100	100	100	100	100	100	100
	N2–H···N7	100	100	100	100	100	100	100
G10 – G15	N1–H···O6	100	100	100	100	100	100	100
	N2–H···N7	99	99	99	100	100	100	100
G11 – G14	N7···H–N2	100	100	100	100	100	100	100
	O6···H–N1	100	100	100	100	100	100	100

[a] Average values are calculated over the entire production MD simulation.

Table S8. Average distances (Å) of hydrogen-bond donor and acceptor atoms in G–tetrads for bound native and modified TBA–thrombin complexes throughout 0.5 μs MD simulations.[a]

Nucleobase Interactions	H–Bond Interactions	Avg. Distances (Å)						
		Native	T3	T4	T7	T9	T12	T13
G1 – G6	N1–H···O6	2.9	3.0	3.0	2.9	3.0	3.0	2.9
	N2–H···N7	3.0	3.0	3.0	2.9	3.0	3.0	3.0
G1 – G15	N7···H–N2	3.0	2.9	3.0	3.0	3.0	3.0	3.0
	O6···H–N1	2.9	2.9	2.9	2.9	2.9	2.9	2.9
G2 – G5	N7···H–N2	3.0	2.9	2.9	3.0	2.9	2.9	2.9
	O6···H–N1	3.0	2.9	2.9	2.9	2.9	2.9	2.9
G2 – G14	N1–H···O6	2.9	2.9	3.0	2.8	3.0	2.9	2.9
	N2–H···N7	3.0	3.0	3.0	3.0	3.0	3.0	3.0
G5 – G11	N7···H–N2	3.0	3.0	3.0	3.0	3.0	3.0	3.0
	O6···H–N1	3.0	3.0	3.0	2.9	3.0	2.9	2.9
G6 – G10	N1–H···O6	2.9	2.9	2.9	2.9	2.9	2.9	2.9
	N2–H···N7	3.0	2.9	2.9	2.9	2.9	2.9	2.9
G10 – G15	N1–H···O6	2.9	2.9	2.9	2.9	2.9	2.9	2.9
	N2–H···N7	3.0	3.0	3.0	3.0	3.0	3.0	3.0
G11 – G14	N7···H–N2	2.9	2.9	3.0	2.9	2.9	2.9	2.9
	O6···H–N1	2.9	2.9	2.9	2.9	2.9	2.9	2.9

[a] Average values are calculated over the entire production MD simulation.

Table S9. Average hydrogen-bond angle (°) in G–tetrads for bound native and modified TBA–thrombin complexes throughout 0.5 μs MD simulations.[a]

Nucleobase Interactions	H–Bond Interactions	Avg. ∠(D–H–A) Angle (°)						
		Native	T3	T4	T7	T9	T12	T13
G1 – G6	N1–H···O6	156	153	152	153	153	152	153
	N2–H···N7	162	162	163	163	162	162	162
G1 – G15	N7···H–N2	161	163	162	158	162	162	161
	O6···H–N1	158	156	156	162	157	157	157
G2 – G5	N7···H–N2	160	162	161	159	162	161	159
	O6···H–N1	157	157	157	160	156	157	159
G2 – G14	N1–H···O6	156	155	155	159	163	156	161
	N2–H···N7	161	164	163	159	154	162	159
G5 – G11	N7···H–N2	162	164	163	161	164	162	159
	O6···H–N1	156	155	157	160	155	158	161
G6 – G10	N1–H···O6	158	156	163	157	163	156	157
	N2–H···N7	160	163	155	162	156	162	162
G10 – G15	N1–H···O6	159	155	155	155	156	155	153
	N2–H···N7	161	163	163	164	163	163	164
G11 – G14	N7···H–N2	160	162	162	161	162	161	158
	O6···H–N1	158	157	158	159	157	159	159

[a] Average values are calculated over the entire production MD simulation.

Table S10. Average K^+ coordination distances (standard deviation in parentheses, Å) in bound native and modified TBA–thrombin complexes throughout 0.5 μs MD simulations.[a]

	Native	T3	T4	T7	T9	T12	T13
K···G1(O6)	2.8 (0.1)	2.8 (0.1)	2.7 (0.1)	2.8 (0.2)	2.8 (0.1)	2.8 (0.2)	2.8 (0.2)
K···G2(O6)	2.8 (0.1)	2.8 (0.1)	2.8 (0.1)	2.8 (0.2)	2.8 (0.1)	2.8 (0.1)	2.7 (0.1)
K···G5(O6)	2.8 (0.2)	2.8 (0.2)	2.8 (0.2)	2.7 (0.1)	2.8 (0.2)	2.8 (0.2)	2.8 (0.2)
K···G6(O6)	2.8 (0.1)	2.8 (0.2)	2.8 (0.2)	2.8 (0.2)	2.8 (0.1)	2.8 (0.2)	2.8 (0.2)
K···G10(O6)	2.7 (0.1)	2.7 (0.1)	2.7 (0.1)	2.7 (0.1)	2.7 (0.1)	2.7 (0.1)	2.8 (0.2)
K···G11(O6)	2.8 (0.2)	2.8 (0.2)	2.8 (0.2)	2.8 (0.2)	2.8 (0.2)	2.8 (0.2)	2.7 (0.1)
K···G14(O6)	2.8 (0.1)	2.8 (0.1)	2.8 (0.2)	2.7 (0.1)	2.8 (0.2)	2.8 (0.1)	2.8 (0.2)
K···G15(O6)	2.8 (0.2)	2.8 (0.2)	2.8 (0.2)	2.8 (0.2)	2.8 (0.2)	2.8 (0.2)	2.8 (0.2)

[a] Average values are calculated over the entire production MD simulation.

Table S11. Average nucleobase–amino acid interactions (standard deviation in parentheses, kcal/mol) in the native and modified TBA–thrombin complexes throughout 0.5 μs MD simulations.[a]

Interactions	Native	T3	T4	T7	T9	T12	T13
G2–Arg97	–2.5 (1.5)	–2.2 (1.5)	–0.5 (1.5)	0.2 (1.8)	–1.8 (2.7)	–0.7 (2.3)	0.4 (1.9)
T3–Ile36	–2.1 (1.1)	–2.5 (0.9)	–2.6 (0.7)	–1.3 (0.7)	–1.5 (1.0)	–1.6 (1.0)	–0.1 (0.2)
T3–His93	–2.1 (1.8)	–1.9 (1.5)	–1.0 (0.9)	0.0 (0.5)	–0.4 (0.8)	–0.7 (1.0)	0.0 (0.0)
T3–Glu99	–4.3 (6.0)	–7.3 (7.8)	3.4 (5.7)	1.7 (1.9)	0.2 (3.5)	–0.8 (4.3)	–3.0 (6.6)
T3–Ile102	–0.9 (1.1)	–1.1 (0.7)	–1.3 (0.7)	–0.9 (0.3)	–2.4 (1.0)	–1.6 (1.0)	–0.1 (0.2)
T3–Tyr141	–1.9 (3.0)	–0.8 (1.7)	–1.0 (1.9)	–0.3 (0.3)	–4.2 (2.8)	–2.1 (2.4)	0.0 (0.2)
T4–Arg97	–22.0 (5.0)	–22.9 (3.0)	–17.4 (5.9)	–12.1 (3.3)	–15.7 (6.9)	–15.0 (7.3)	–8.4 (5.8)
T4–Arg100	–16.1 (4.5)	–16.1 (4.7)	–15.7 (1.6)	–17.9 (1.2)	–15.2 (3.9)	–17.5 (2.2)	–7.6 (8.5)
T4–Asn101	0.1 (0.3)	–0.2 (1.1)	0.1 (0.4)	–0.2 (1.3)	–0.6 (1.8)	–0.1 (1.2)	0.1 (0.2)
G5–Arg100	–11.8 (2.6)	–12.0 (2.1)	–12.2 (1.7)	–12.7 (2.7)	–12.4 (2.6)	–12.6 (2.3)	–3.8 (4.2)
G11–Arg100	–0.7 (2.0)	–0.8 (2.1)	0.3 (1.3)	–1.7 (1.5)	–1.3 (2.0)	–0.9 (2.0)	0.9 (1.0)
T12–Arg89	–5.4 (6.8)	–9.3 (6.2)	–8.1 (5.8)	–4.7 (2.7)	–5.0 (2.7)	–3.0 (2.0)	–4.4 (6.0)
T12–Tyr98	–4.4 (1.0)	–4.8 (1.7)	–4.9 (1.9)	–5.2 (1.9)	–5.5 (1.4)	–5.7 (1.6)	–3.8 (1.7)
T13–Arg97	–17.2 (2.3)	–16.1 (2.3)	–14.1 (5.4)	–19.1 (2.9)	–18.9 (3.5)	–17.5 (4.2)	–12.2 (4.5)
T13–Tyr98	–2.9 (0.6)	–2.8 (0.6)	–2.9 (0.6)	–2.0 (0.4)	–2.5 (0.6)	-2.5 (0.7)	–4.4 (1.9)
T13–Arg100	–11.9 (4.7)	–11.9 (5.1)	–9.3 (1.2)	–9.3 (0.9)	–11.4 (4.4)	–9.4 (1.9)	–3.3 (2.1)
G14–Arg97	–15.7 (2.7)	–15.8 (3.6)	–14.6 (3.3)	–11.5 (2.9)	–14.4 (4.3)	–12.5 (4.5)	–9.8 (4.0)

[a] Interaction strengths were calculated using B3LYP-D3(BJ)/6-311+G(2df,p) single-point calculations on structures taken at 5 ns intervals throughout the 0.5 μs MD simulation. See computational methods section in the main text for full details.

Table S12. Average nucleobase stacking interactions (standard deviation in parentheses, kcal/mol) in the bound native and modified TBA–thrombin complexes throughout 0.5 μs MD simulations.[a]

Bases	Native	T3	T4	T7	T9	T12	T13
G2–T4	–8.1 (1.4)	–8.0 (1.5)	–9.8 (1.3)	–7.5 (1.2)	–8.3 (1.4)	–7.4 (1.6)	–7.4 (1.7)
T3–T4	–0.9 (1.5)	–1.9 (1.3)	–4.7 (1.4)	–1.2 (0.5)	–0.3 (0.9)	–0.6 (0.9)	–1.5 (1.1)
T4–G5	–1.9 (0.7)	–2.2 (1.1)	–1.0 (0.8)	–F1.0 (1.3)	–1.6 (0.7)	–1.7 (1.0)	–2.9 (1.2)
G6–T7	–7.3 (1.0)	–7.3 (1.2)	–7.3 (1.3)	–8.1 (1.3)	–7.4 (1.1)	–7.4 (1.1)	–6.8 (2.2)
G8–G10	–6.8 (1.2)	–6.9 (1.3)	–6.8 (1.3)	–7.0 (1.0)	–6.5 (1.1)	–6.9 (1.2)	–7.3 (1.4)
G11–T13	–8.2 (1.4)	–7.8 (1.5)	–7.6 (1.8)	–5.8 (1.2)	–6.6 (2.3)	–6.6 (1.8)	– 9.6 (1.7)
T13–T14	–1.6 (0.7)	–1.7 (0.8)	–1.7 (0.7)	–2.3 (0.6)	–2.1 (0.8)	–2.1 (0.7)	–2.8 (1.5)
T12–T13	–	–	–	–	–	–	–5.0 (1.6)

[a] Stacking interactions were calculated using B3LYP-D3(BJ)/6-311+G(2df,p) single-point calculations on structures taken at 5 ns intervals throughout the 0.5 μs MD simulation. See computational methods section in the main text for full details.

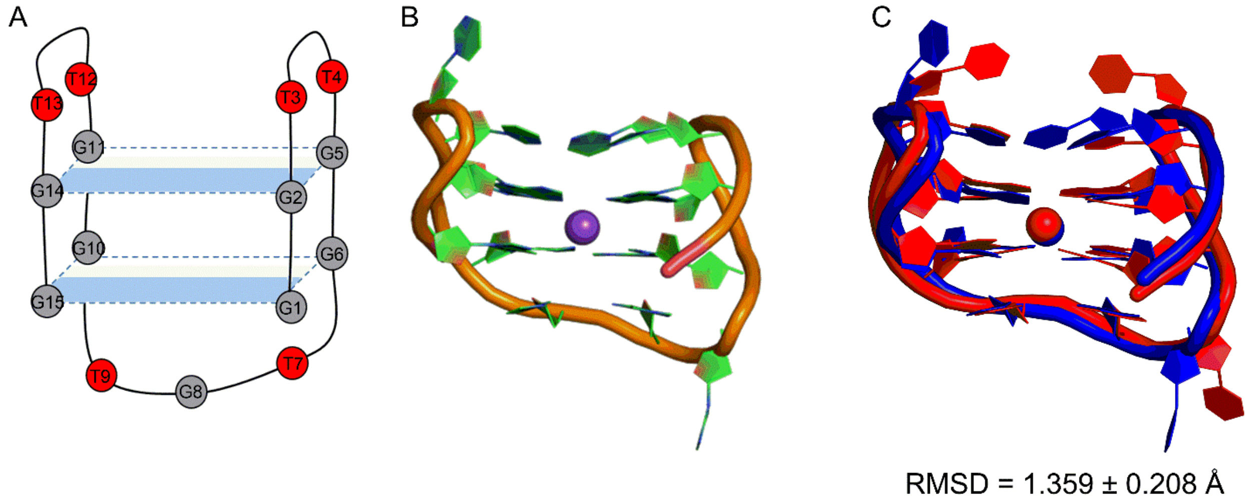

Figure S1. (A) Schematic representation of TBA, with modification sites highlighted in red. (B) MD representative structure of native TBA. (C) Overlay of DNA aptamer from crystal (PDB ID: 4DII, red) and MD representative (blue) structures of unbound native TBA. Average backbone RMSD (Å) with respect to the crystal structure was calculated over the 0.5 μs MD simulation.

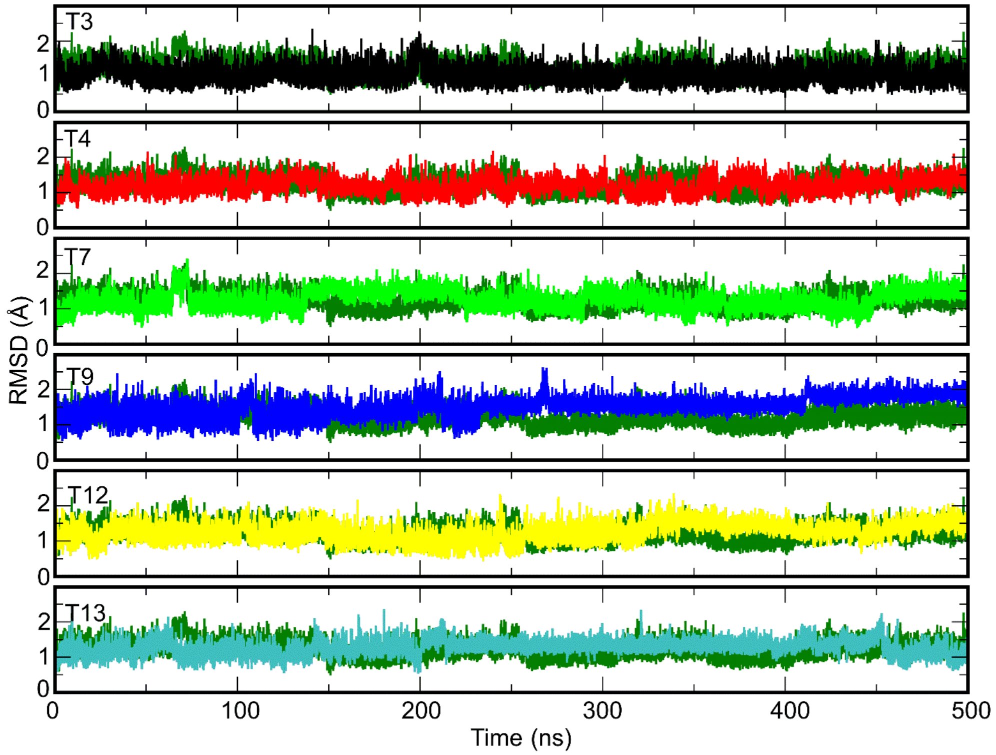

Figure S2. Structural deviations in the backbone (RMSD, Å) with respect to the first simulation frame for each modified TBA compared to native TBA (dark green) throughout 0.5 μs MD simulations.

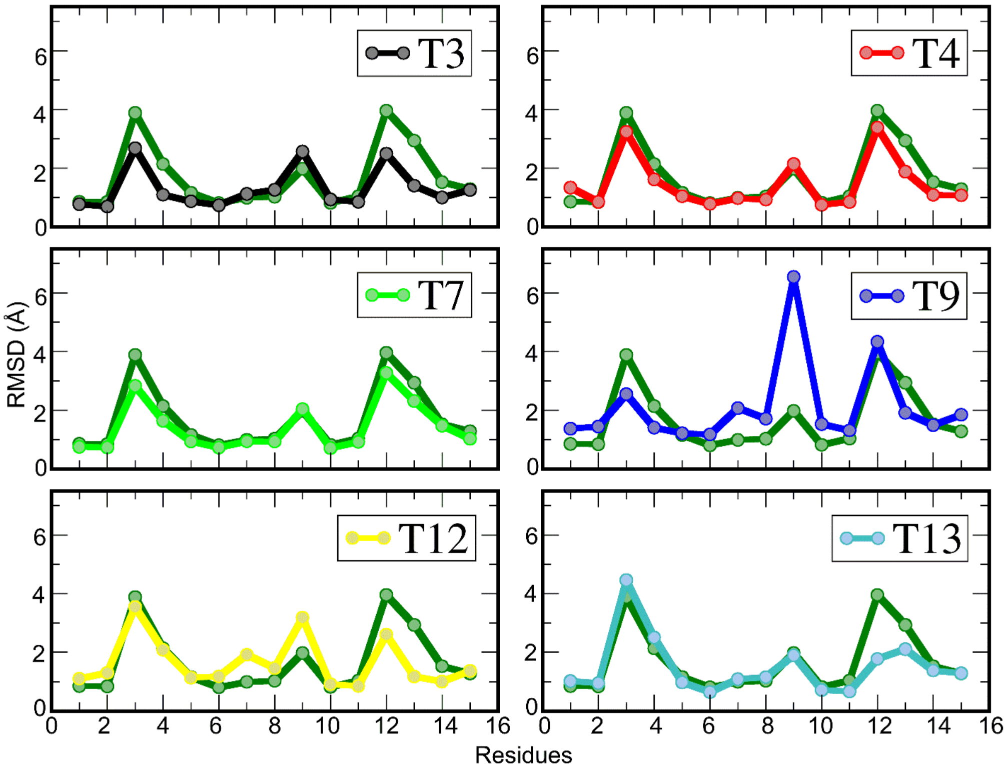

Figure S3. Average structural deviation in each nucleotide (RMSD, Å) with respect to the first simulation frame for each modified TBA compared to native TBA (dark green) throughout 0.5 μs MD simulations.

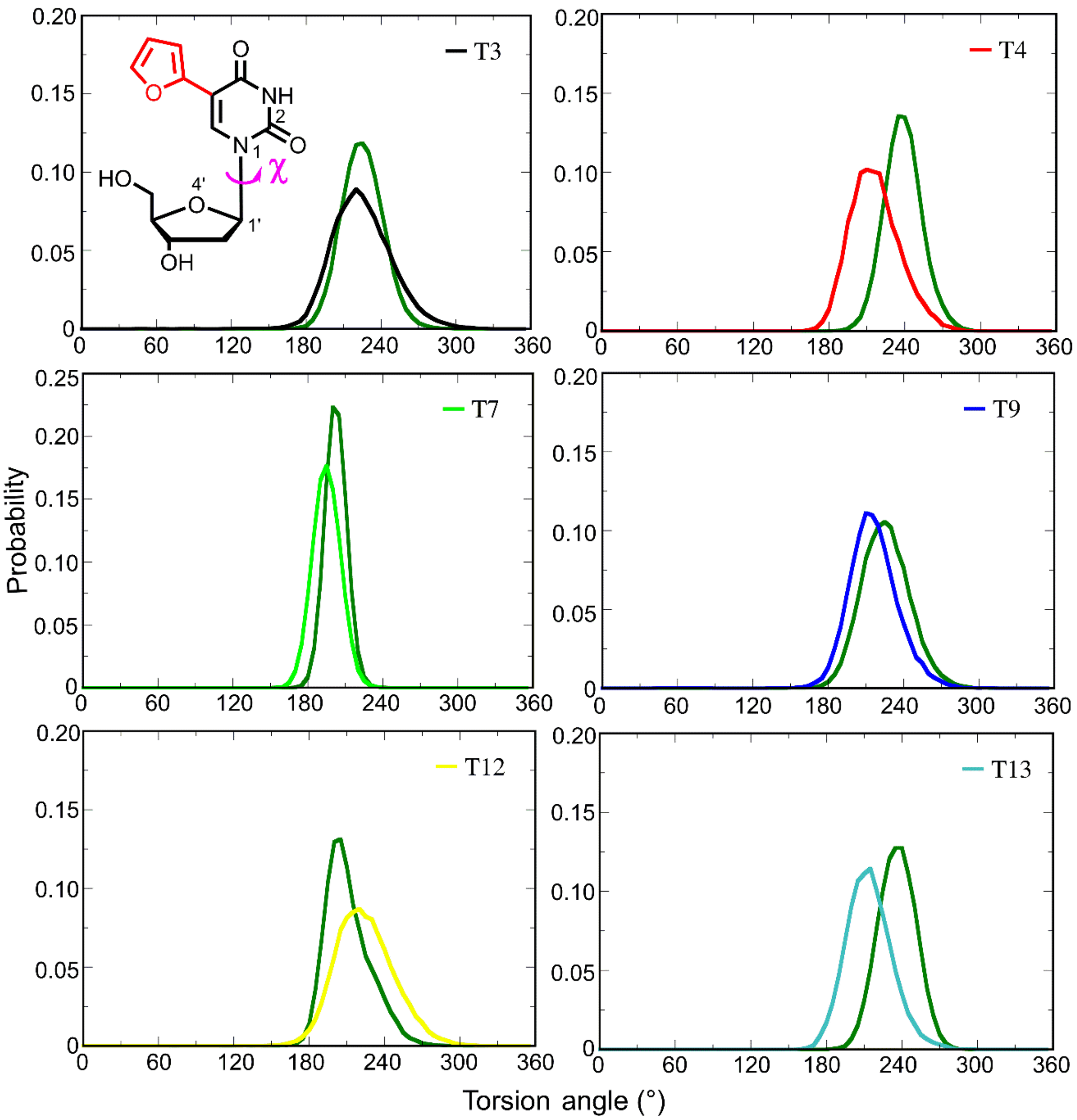

Figure S4. Probability distribution in the χ torsion angle (∠(O4′C1′N1C2), degrees) for the base at position T3, T4, T7, T9, T12 or T13 in native (dark green) and modified TBA. Chemical structure of the modified base (5FurU) is shown in the top left graph.

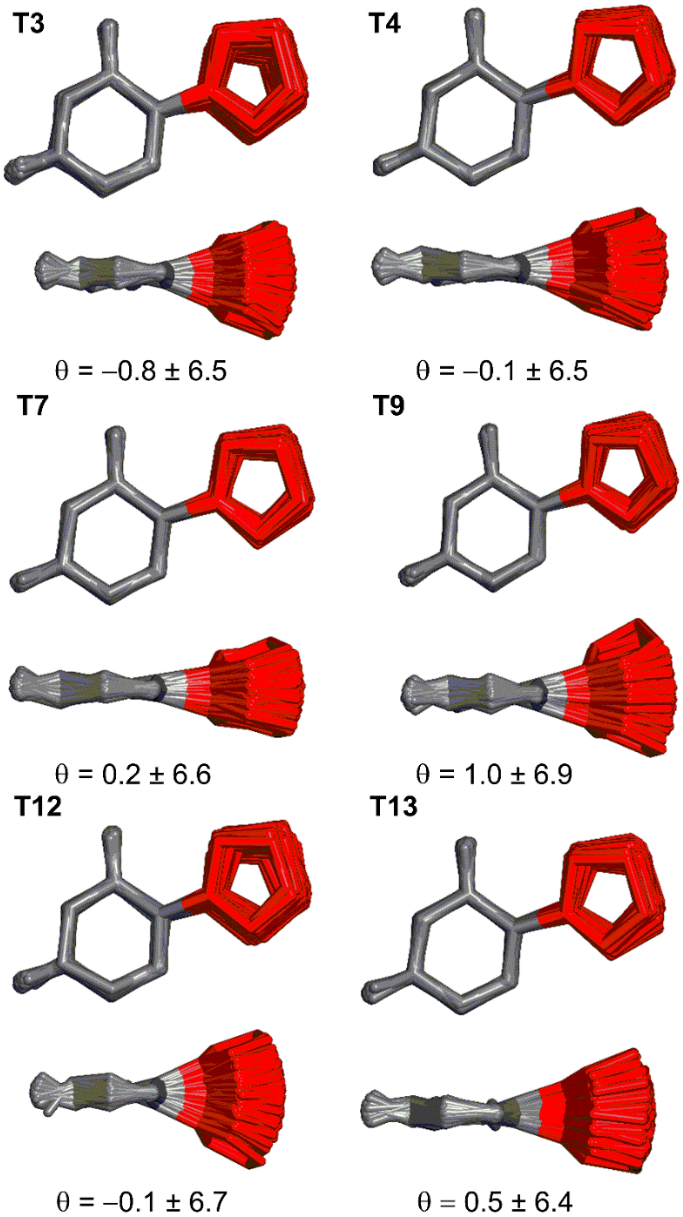

Figure S5. Overlay of the modified 5FurU nucleobase with respect to nucleobase ring for different modified TBA. 5-furyl moiety is shown in red. Average θ torsion angle (∠(C6C5C7O8)) and standard deviation (degrees) calculated throughout the 0.5 μs MD simulations.

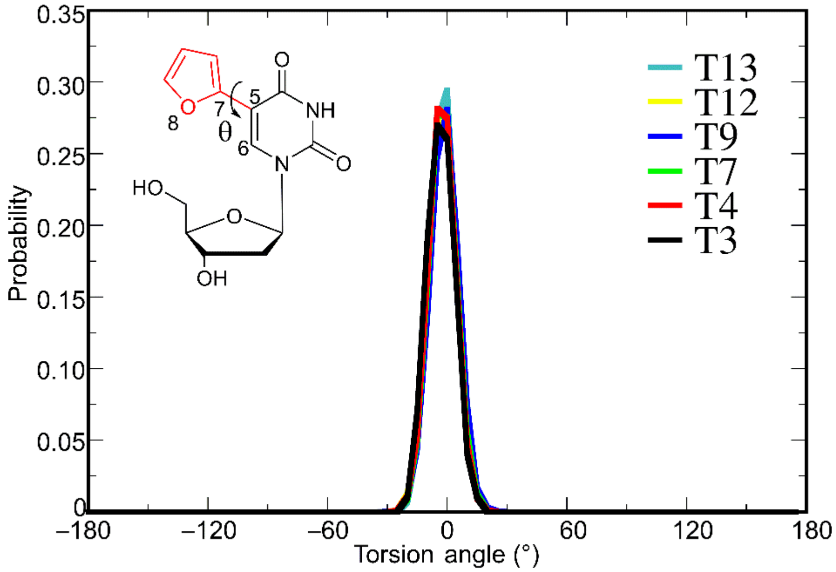

Figure S6. Probability distribution in the θ torsion angle of 5FurU (∠(C6C5C7O8), degrees) for each modified TBA calculated over the 0.5 μs MD simulations. Chemical structure of the modified base (5FurU) is shown in the top left.

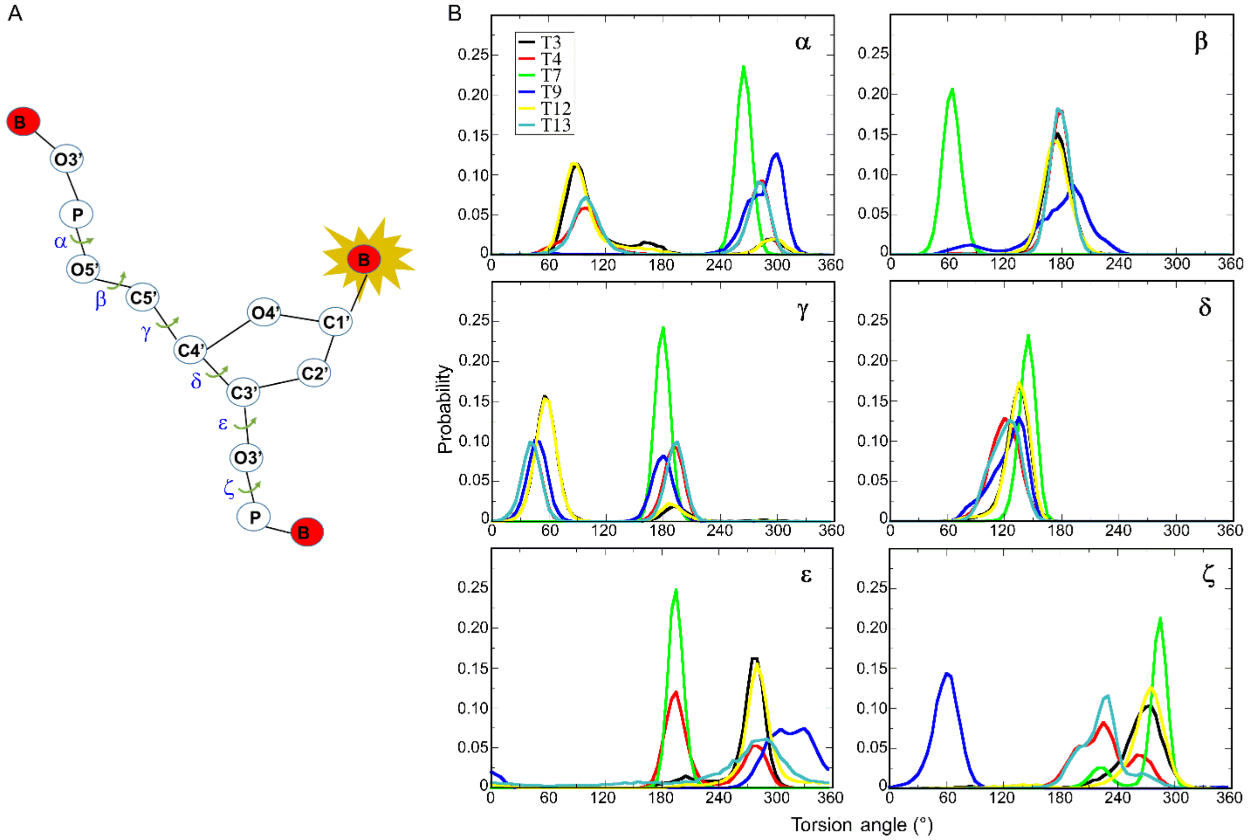

Figure S7. (A) Definition of nucleotide backbone torsion angles (α, β, γ, δ, ε, and ζ). (B) Probability distribution in the nucleotide backbone torsion angles in the modified 5FurU base at each TBA position.

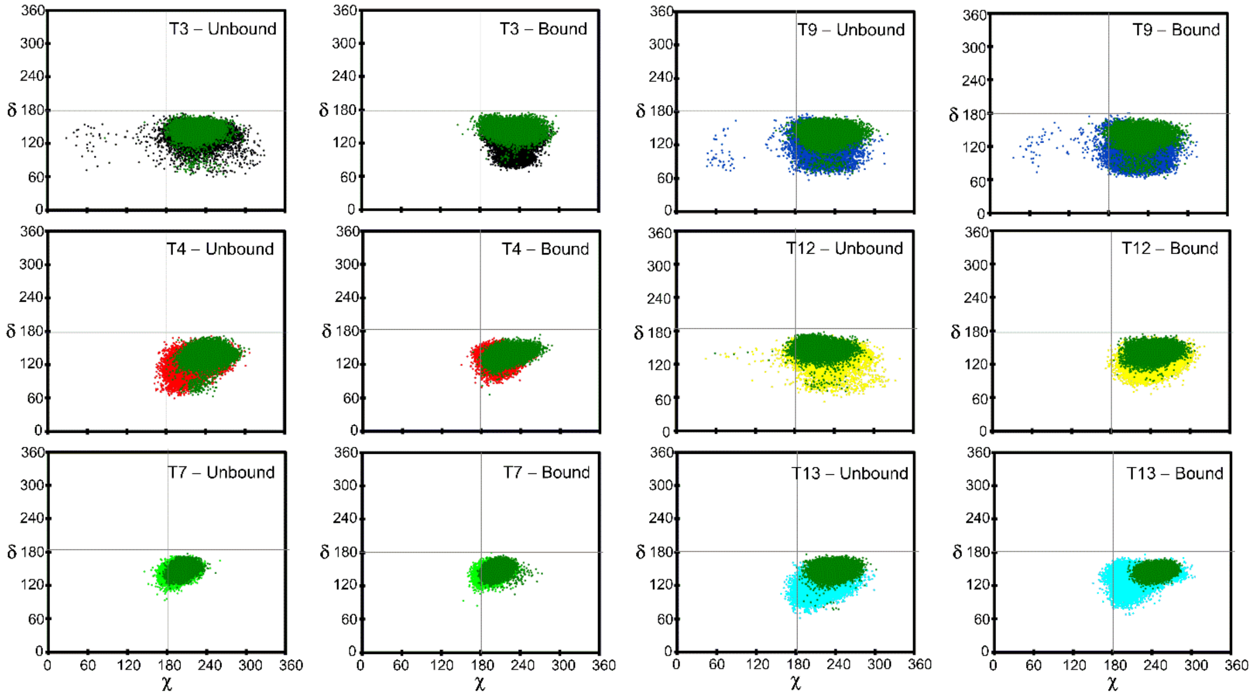

Figure S8. Scatter plot of the χ (∠(O4′C1′N1C2)) versus δ (∠(C5′C4′C3′O3′)) torsion angles for the native (dark green) and modified base at various positions in unbound (left) and bound (right) TBA.

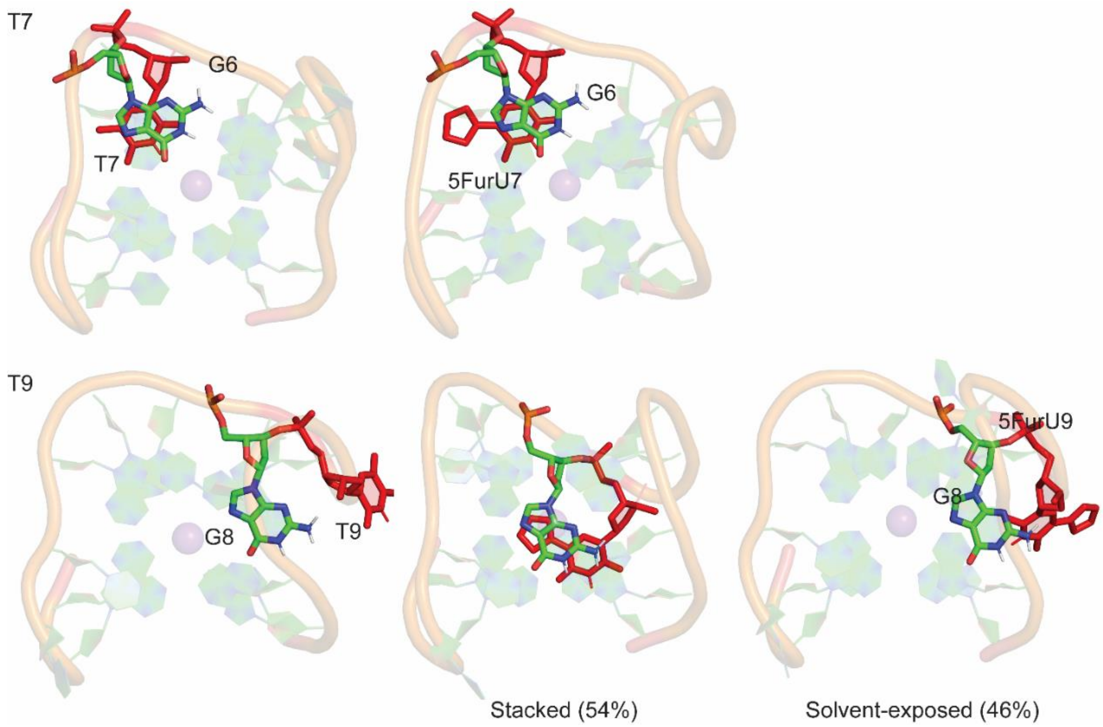

Figure S9. Stacking interactions of the T7 (top, red) and T9 (bottom, red) bases in unbound native (left) and modified (middle and right) TBA aptamer.

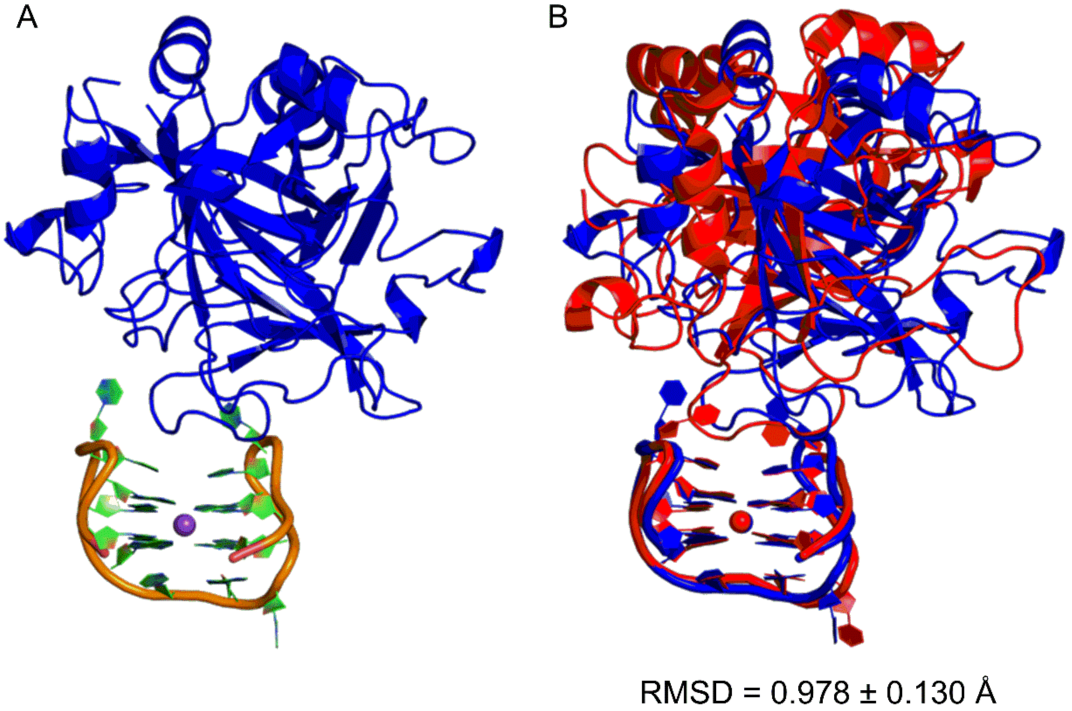

Figure S10. (A) MD representative structure of the native TBA–thrombin complex. (B) Overlay of crystal (PDB ID: 4DII, red) and MD representative (blue) structures of TBA–thrombin complex. Average backbone RMSD with respect to the crystal structure (Å) was calculated over the 0.5 μs MD simulation.

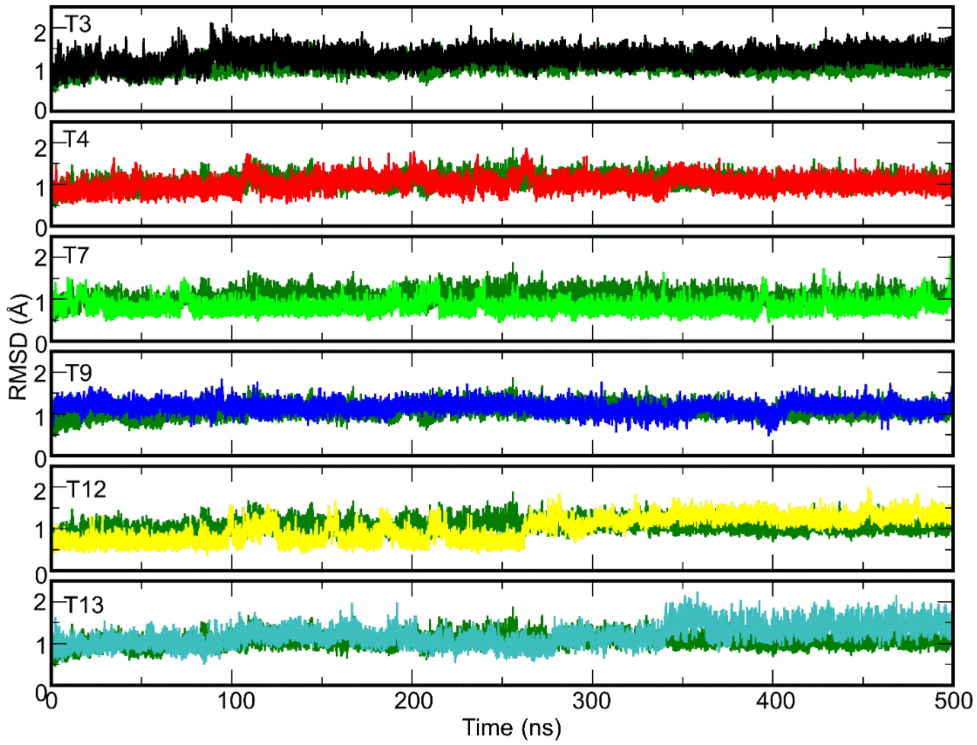

Figure S11. Structural deviations in the backbone (RMSD, Å) with respect to the first simulation frame for native (dark green) and modified TBA–thrombin complexes throughout 0.5 μs MD simulations.

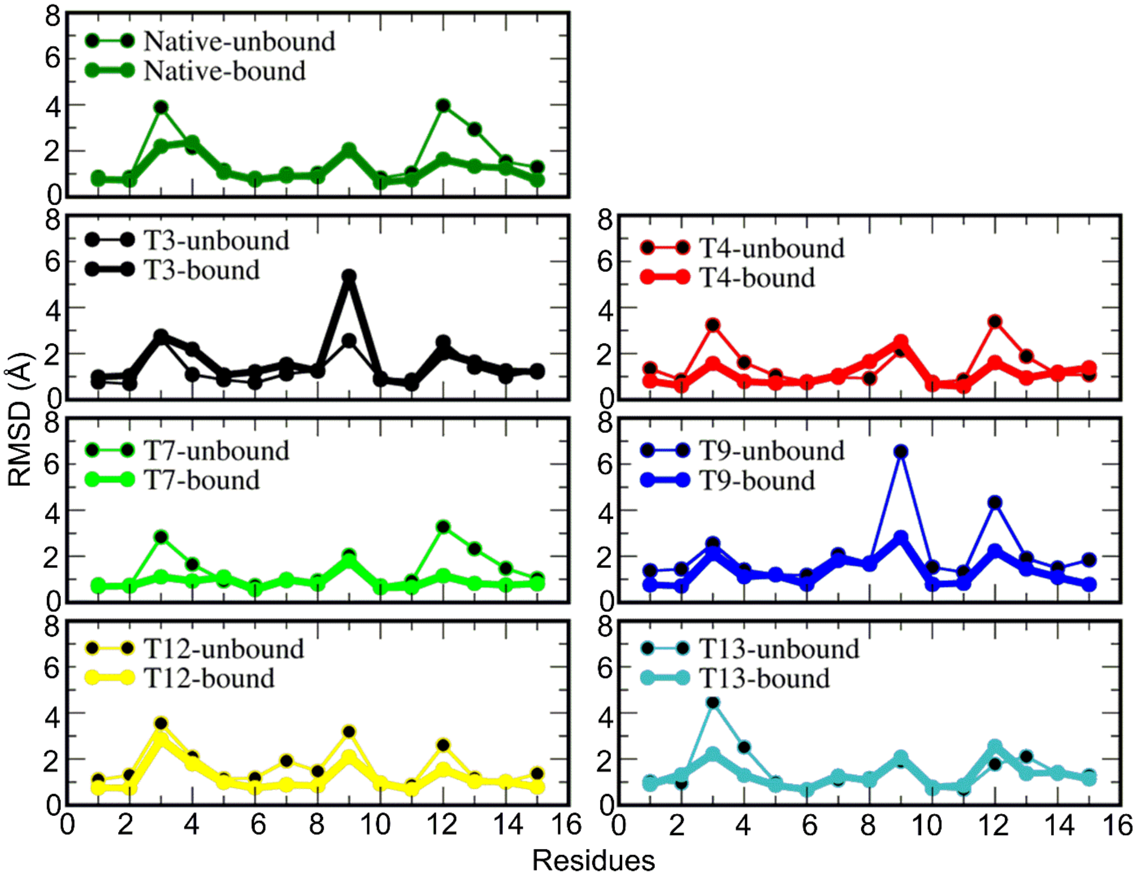

Figure S12. Average structural deviations (RMSD, Å) with respect to the first simulation frame for each nucleotide in native and modified unbound TBA (black circles) compared to the corresponding TBA–thrombin complex (colored circles) throughout the 0.5 μs MD simulations.

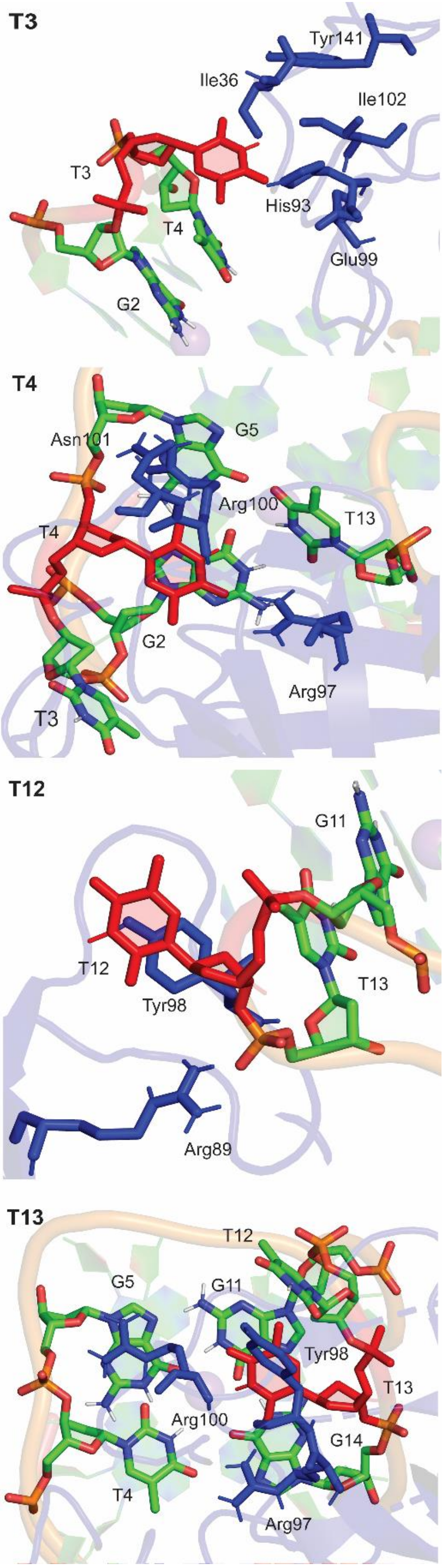

Figure S13. Nucleobase (green) and amino acid (blue) residues surrounding the T base at the T3, T4, T12 or T13 position (red) at the DNA–protein interface in native TBA–thrombin complexes.

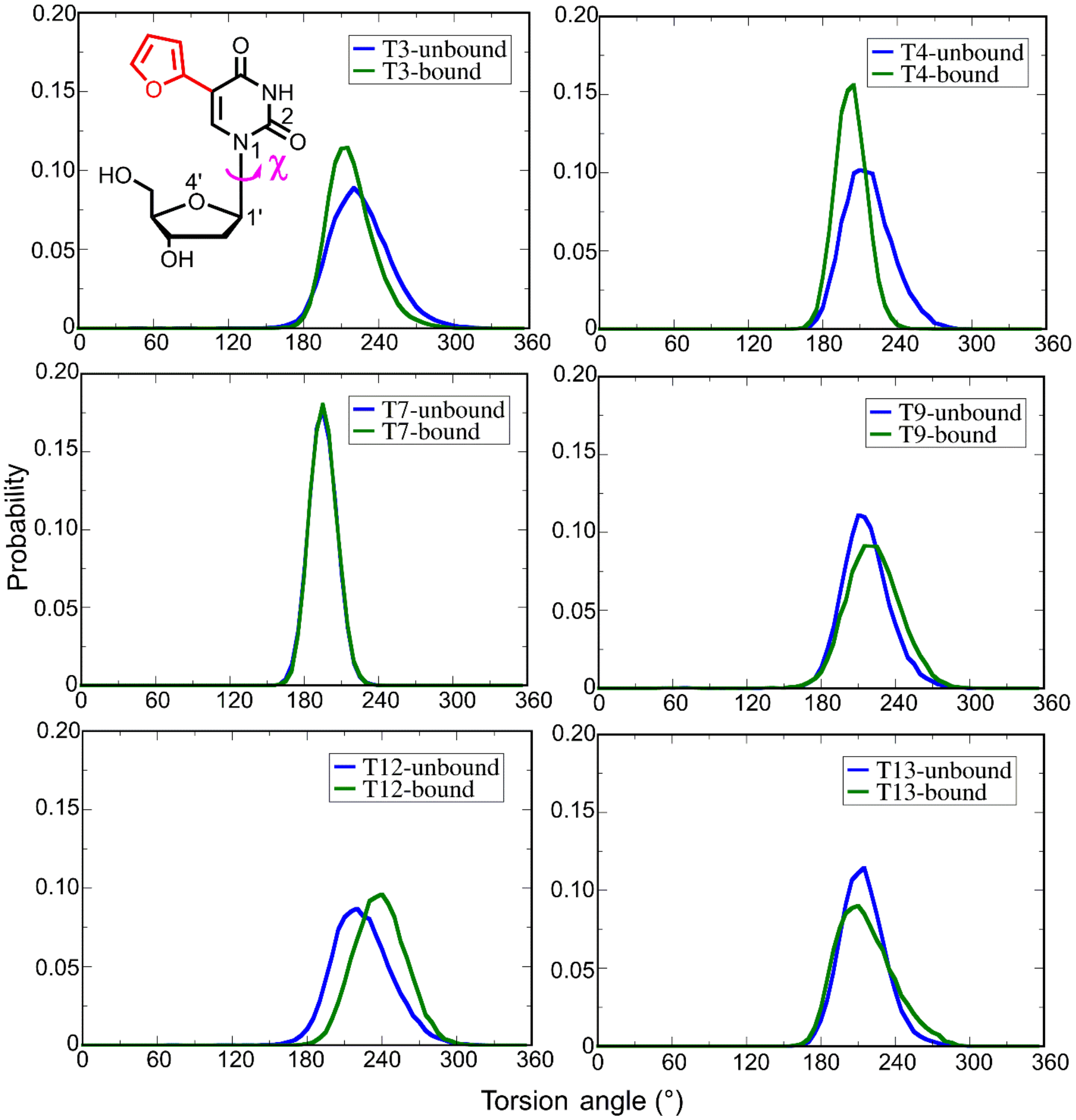

Figure S14. Probability distribution in the χ torsion angle (∠(O4′C1′N1C2), degrees) for the modified base at position T3, T4, T7, T9, T12 or T13 in the modified TBA–thrombin complex compared to the native TBA–thrombin complex (dark green) calculated over the 0.5 μs MD simulations. Chemical structure of the modified base (5FurU) is shown in the top left graph.

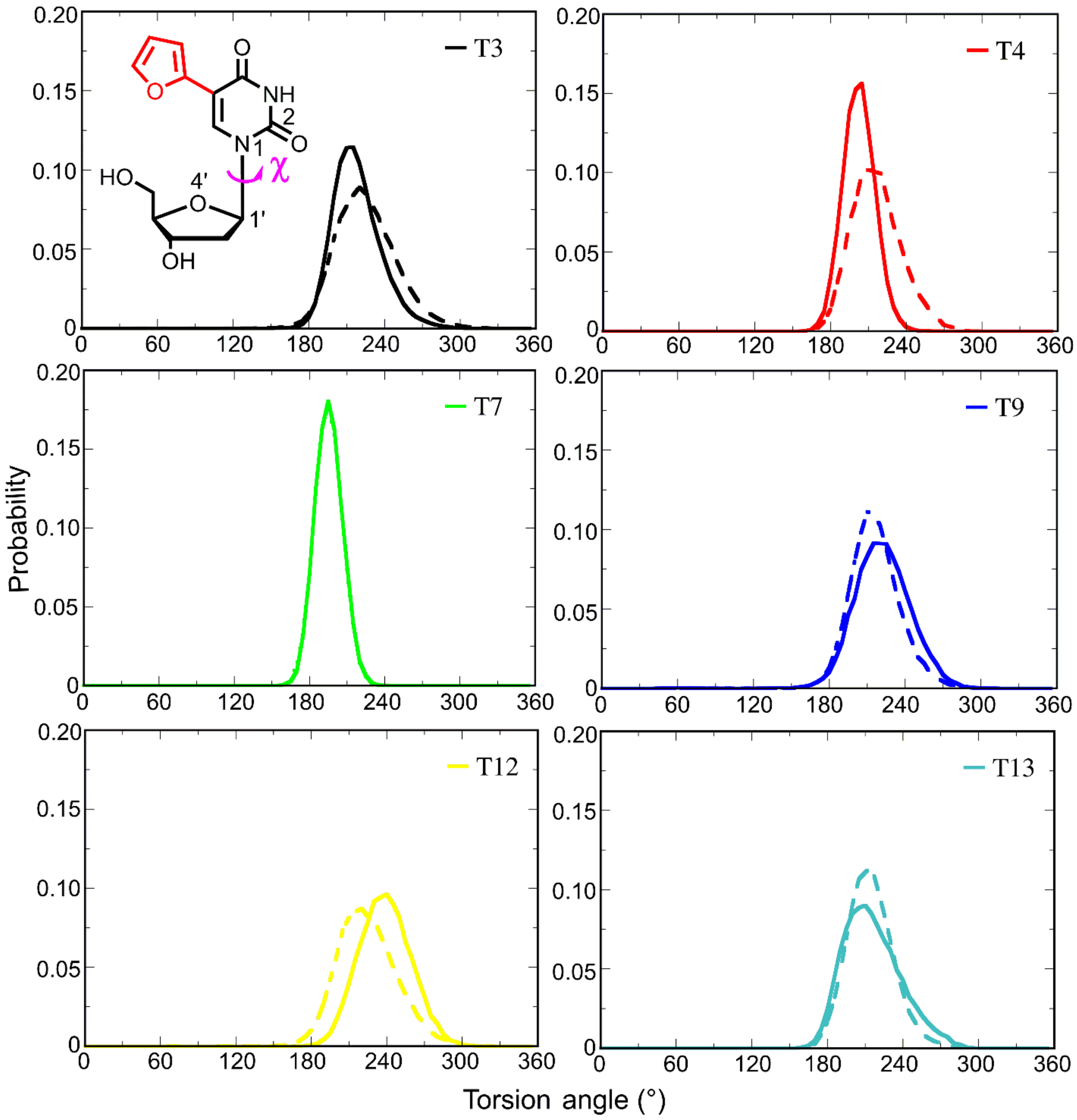

Figure S15. Probability distribution in the χ torsion angle (∠(O4′C1′N1C2), degrees) for the modified base at position T3, T4, T7, T9, T12 or T13 in TBA (dotted line) and the TBA–thrombin complex (line) calculated over the 0.5 μs MD simulation. Chemical structure of the modified base (5FurU) is shown in the top left graph.

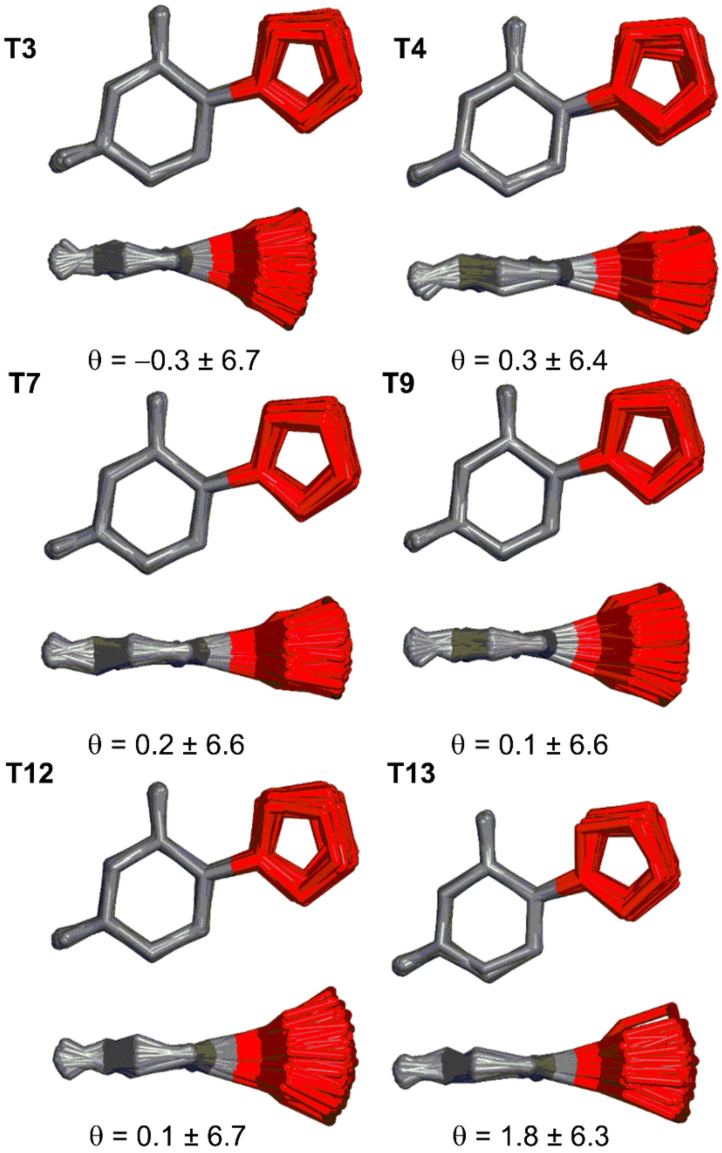

Figure S16. Overlay of the modified 5FurU nucleobase with respect to nucleobase ring for different modified TBA–thrombin complexes. 5-Furyl moiety is shown in red. Average θ torsion angle (∠(C6C5C7O8)) and standard deviation (degrees) calculated throughout the 0.5 μs MD simulations.

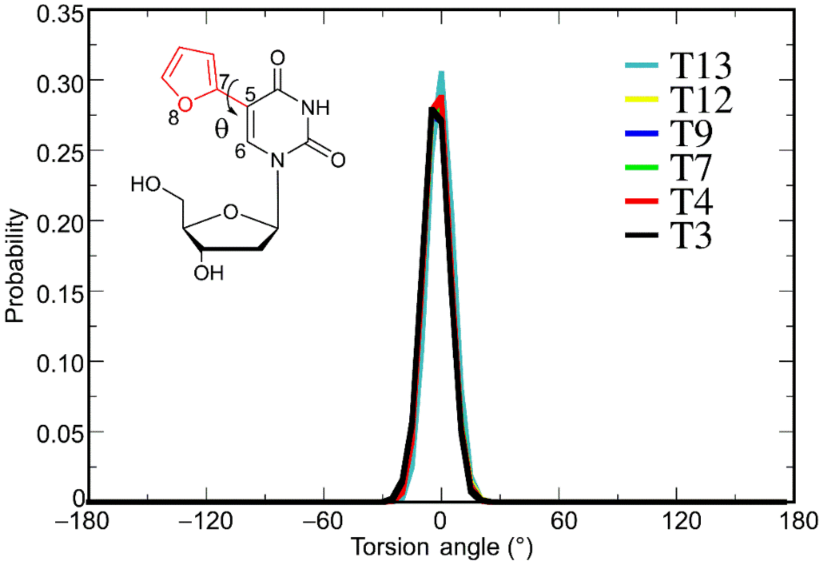

Figure S17. Probability distribution in the θ torsion angle (∠(C6C5C7O8), degrees) for each modified TBA–thrombin complex calculated over the 0.5 μs MD simulations. Chemical structure of the modified base (5FurU) is shown in the top left.

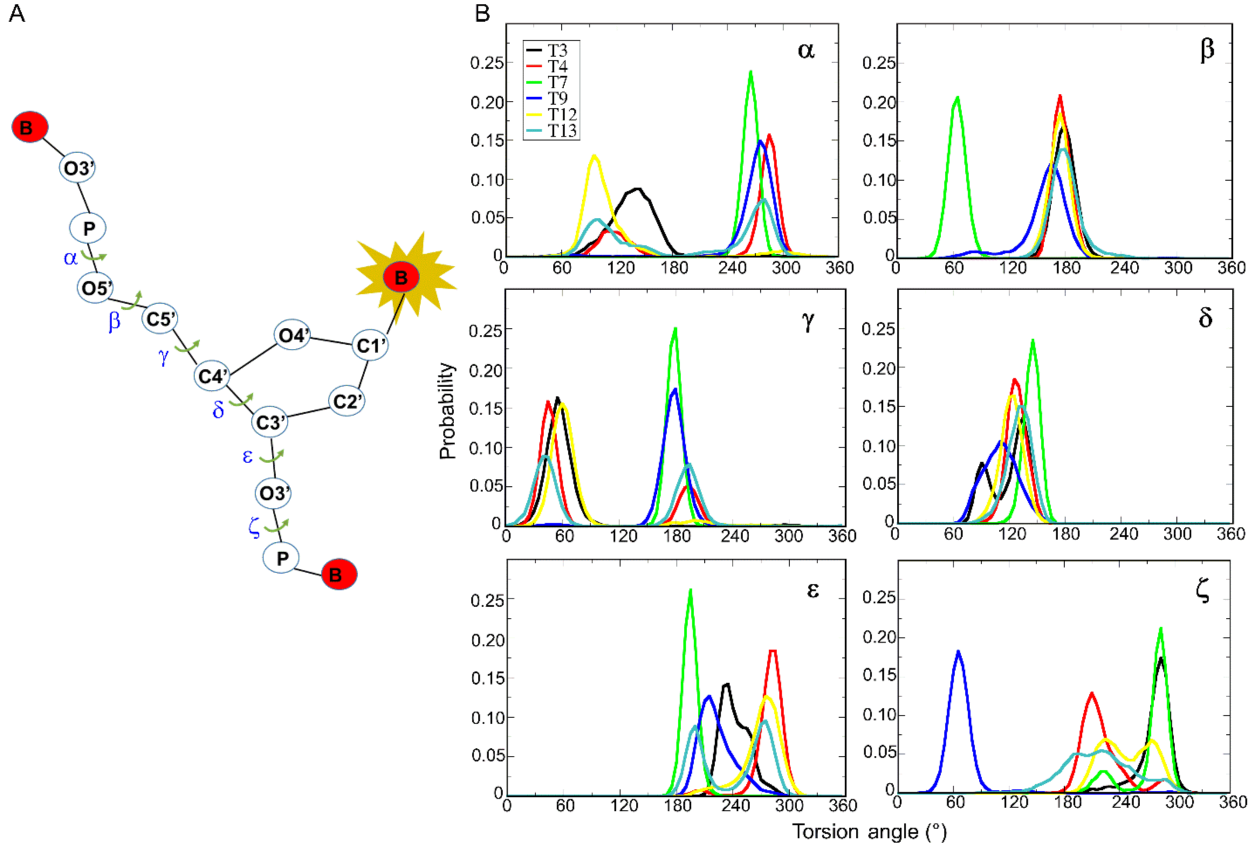

Figure S18. (A) Definition of nucleotide backbone torsion angles (α, β, γ, δ, ε, and ζ). (B) Probability distribution in the nucleotide backbone torsion angles in the modified 5FurU base at each position in TBA–thrombin complexes.
